# Selection of a picomolar antibody that targets CXCR2-mediated neutrophil activation and alleviates EAE symptoms

**DOI:** 10.1038/s41467-021-22810-z

**Published:** 2021-05-05

**Authors:** Xiaojie Shi, Yue Wan, Nan Wang, Jiangchao Xiang, Tao Wang, Xiaofeng Yang, Ju Wang, Xuxue Dong, Liang Dong, Lei Yan, Yu Li, Lili Liu, Shinchen Hou, Zhenwei Zhong, Ian A. Wilson, Bei Yang, Guang Yang, Richard A. Lerner

**Affiliations:** 1 Shanghai Institute for Advanced Immunochemical Studies, ShanghaiTech University, Shanghai, China; 2 School of Life Science and Technology, ShanghaiTech University, Shanghai, China; 3 University of Chinese Academy of Sciences, Beijing, China; 4 CAS Center for Excellence in Molecular Cell Science, Shanghai Institute of Biochemistry and Cell Biology, Chinese Academy of Sciences, Shanghai, China; 5 Department of Integrative Structural and Computational Biology, The Scripps Research Institute, La Jolla, California, CA USA; 6 The Skaggs Institute for Chemical Biology, The Scripps Research Institute, La Jolla, La Jolla, CA USA; 7 Department of Chemistry, The Scripps Research Institute, La Jolla, CA USA

## Abstract

Receptors and their ligands are important therapeutic targets for about one third of marketed drugs. Here, we describe an epitope-guided approach for selection of antibodies that modulate cellular signaling of targeted receptors. We chose CXC chemokine receptor 2 (CXCR2) in the G-protein coupled receptor superfamily as receptor and a CXCR2 N-terminal peptide for antibody selection. We obtain a highly selective, tight-binding antibody from a 10^11^-member antibody library using combinatorial enrichment. Structural and Hydrogen-Deuterium-Exchange mass spectrometry analyses demonstrate antibody interaction with an N-terminal region of CXCR2 that is part of the IL-8 epitope. The antibody strongly inhibits IL-8-induced and CXCR2-mediated neutrophil chemotaxis in vitro and alleviates *h*CXCR2-dependent experimental autoimmune encephalomyelitis symptoms in mice. As inappropriate neutrophil migration accompanies many diseases including inflammatory bowel disease, glomerulonephritis, allergic asthma, chronic obstructive pulmonary disease, and cancer, this antibody has potential for development as a therapeutic agent, akin to anti-TNF antibodies. However, an important difference here is that the antibody targets the chemokine receptor and competes with natural ligand, rather than targeting the ligand itself.

## Abstract (web summary)

CXCR2 is central to neutrophil chemotaxis and hence to some inflammatory diseases. Here the authors demonstrate the value of an epitope-guided antibody panning method to develop a tight binding anti-hCXCR2 antibody, along with crystal structures of this antibody and antigen, that can block neutrophil chemotaxis and protect mice in an EAE model.

## Introduction

The G protein coupled-receptor (GPCR) superfamily is one of the largest families in the human genome, consisting of over eight hundred members, with a broad spectrum of distribution in various organs and tissues, including the central nervous, immune, and cardiovascular systems. GPCRs are widely involved in a range of physiological and pathological processes in humans. Almost 40% of approved drugs mediate their effects through GPCRs^1^. Based on evolutionary homology and properties of their physiological ligands, most GPCR proteins can be grouped into one of five main families: *Rhodopsin*, *Adhesion*, *Secretin*, *Glutamate* and *Frizzled/TAS2*^2^. *Rhodopsin* is the largest and most heterogeneous family, and can be further divided into four sub-families, α, β, γ, and δ^2^. These proteins have a similar overall structure: an extracellular N-terminal region, seven transmembrane helices, and a cytoplasmic C-terminal domain. GPCRs sense various extracellular stimuli through interactions with a variety of ligands, such as amino acids, nucleic acids, peptides and proteins, and activate intracellular signaling pathways *via* ligand-induced conformational changes. In canonical signaling, GPCR signals are transduced through recruitment of intracellular GTP-dependent proteins (G proteins) onto specific cytoplasmic regions of the C-terminus - the so-called G protein-dependent signaling. Then, depending on the particular G protein involved, downstream pathways including cAMP or the PIP2 pathway are activated^3^. Moreover, recruitment of other intracellular scaffold proteins, e.g., β-arrestins, has been shown to activate G protein-independent signaling events^4^. Multiplex signaling endows GPCRs with parallel functions in a cell, making it difficult to design relevant assays linking any single target receptor to specific downstream cellular activities. It remains a challenge for the pharmaceutical industry to develop highly effective therapeutics against these important targets. In addition, while antibodies have emerged as an ever increasing source of new therapies, as yet only two therapeutic antibodies to GPCRs have been developed (to CCR4 and CGRPR)^5,6^, although others have been generated, including to CXC chemokine receptor 1 (CXCR1) and CXCR2, but not advanced to therapy^7–10^. This paucity is largely due to the difficulty in generating antibodies that bind to functional conformations of these membrane proteins.

Combinatorial antibodies have emerged as a powerful tool in drug discovery^11,12^. Over 80 antibodies from phage panning have entered clinical studies, and more than 10 have been granted marketing approval^13^. The combinatorial antibody library approach takes advantage of vast diversity consisting of up to 10^14^ distinct binding molecules^14^. Antibodies selected from such libraries have shown diverse mechanisms, and can be neutralizing or function as agonists, antagonists, or inverse agonists and, in some cases, function beyond the scope of native ligands^15–17^.

CXCR2, a member of the chemokine receptor family, is mainly expressed on neutrophils^18^. CXCR2 is involved in neutrophil chemotaxis, which normally follows inflammatory stimuli^19–23^. However, unwanted migration of neutrophils can also be a factor in the pathophysiology of a wide variety of diseases associated with inflammation, including colitis, chronic obstructive pulmonary disease (COPD), asthma, and glomerulonephritis^24–26^. For example, a previous study has reported that depletion of CXCR2 protects lungs from cigarette smoke-induced inflammation and injury^27^. Multiple sclerosis (MS), an autoimmune disease of demyelination in the central nervous system with strong inflammation, was previously thought to be caused mainly by the overreaction of T (T_H_1 & T_H_17) and B cells (autoimmune antibody). Current emerging evidences revealed the important role and involvement of neutrophils and CXCR2 signaling in MS^28,29^. In addition, CXCR2 has been shown to participate in the progression of different types of cancer, playing a significant role in proliferation, survival, and metastasis of tumor cells, and affecting the whole tumor microenvironment^30,31^.

Given these clinical findings, inhibition of CXCR2-induced neutrophil migration is an important, but as yet unrealized, strategy to treat neutrophil-related inflammatory diseases and some cancers^32–34^. Several small molecules targeting CXCR2 have already shown remarkable inhibitory effect in both in vitro and animal studies, but not therapeutic efficacy in the clinical setting^32,33^. This lack of clinical effectiveness is thought to be due to non-functional binding to CXCR2 and off-target effects^35^. In contrast, antibodies have the potential to overcome these clinical difficulties in that they have high specificity, high serum stability, high safety and, because of their size, can target the ligand-binding extracellular domains of receptors. However, because of the difficulty in preparing adequate quantities of stable antigen in native conformation for selection, it has been challenging to identify functional antibodies targeting GPCRs.

Here, using an epitope-guided approach, we selected a combinatorial antibody from a 10^11^-member library that binds CXCR2 in the same epitope region as IL-8 (also known as CXCL8), but with picomolar potency. The superior antibody affinity compared with IL-8 (picomolar *vs*. nanomolar) overcomes the competition problem and results in total inhibition of IL-8-induced cellular functions, thus indicating potential for development as a therapeutic modality for treatment of neutrophil-related diseases. The selection strategy might also be generalized to develop antibodies for other GPCR-related diseases.

## Results

### Selection of combinatorial antibodies that bind human CXCR2

**Fig. 1:**
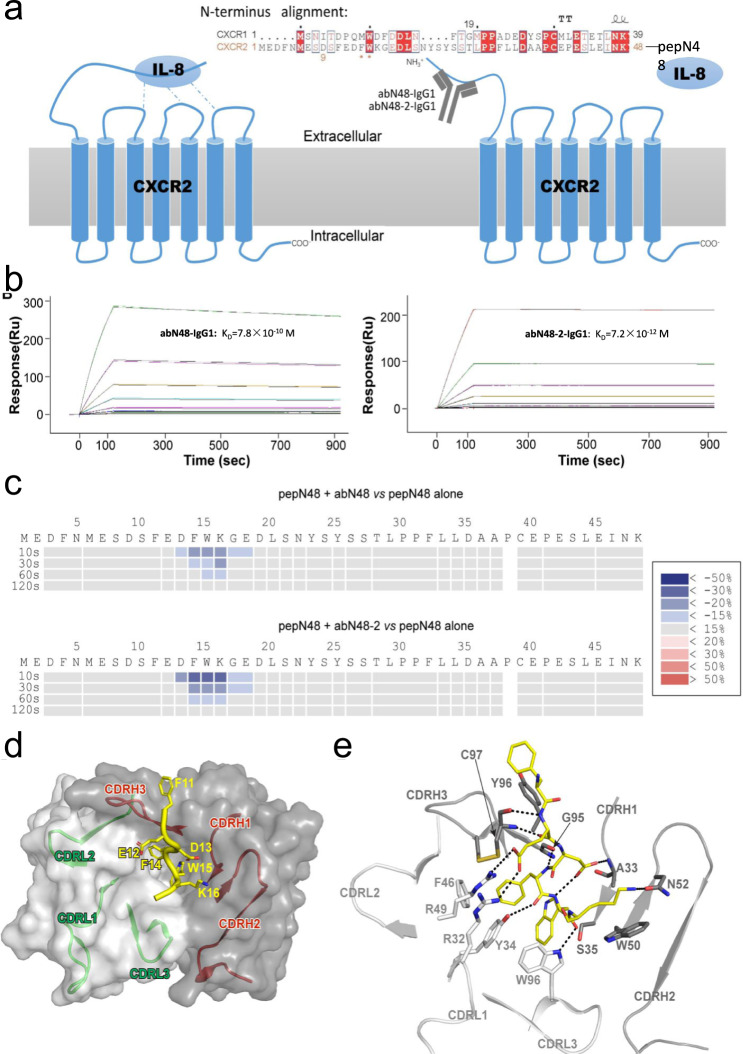
Epitope-guided selection of tight binding antibodies for *h*CXCR2. **a** Schematic illustration of antibodies selected by phage panning based on the IL-8 epitope on the extracellular N-terminus of *h*CXCR2 (pepN48). **b** Binding affinity measurements of two combinatorial antibodies, abN48-IgG1 and abN48-2-IgG1 by SPR assay on a Biacore T200. Curves in the sensorgram plots represent a range of concentrations of antibody solutions: 0.1, 0.5, 1, 2, 5 and 10 nM for abN48-IgG1 and 0.1, 0.2, 0.5, 1, 2, and 5 nM for abN48-2-IgG1. **c** HDX exchange for pepN48 in the presence of abN48-IgG1 (upper panel) or abN48-2-IgG1 (lower panel) as compared to free pepN48. The percentage difference in deuterium uptake values at different labeling time points is represented as heatmap, with light gray indicating no significant changes, red indicating increase, and blue indicating decrease in HDX exchange rates. **d** Crystal structure of pepN9-19 in complex with abN48-2 illustrating that the aromatic side chains of Phe14 and Trp15 from pepN9-19 snugly fit into a cavity formed by the antibody CDR loops. The light and heavy chains of abN48-2 are shown as light and dark gray surfaces, respectively. The CDR loops of the heavy chain and light chain are depicted as red and green cartoons respectively and the surface representation was rendered transparent to show the location of the CDR loops. The backbone of bound pepN9-19 is depicted as a yellow tube with the side chains of critical residues as stick models. **e** Close-up view of the interface between pepN9-19 and abN48-2. Key residues involved in the antibody-antigen interface are shown as stick models and color-coded as following: light gray (light chain residues), dark gray (heavy chain residues), and yellow (pepN9-19 residues). Polar and electrostatic interactions are indicated with black dashed lines. The CDRs residues are labeled following Kabat numbering.

The human CXCR2 (*h*CXCR2) extracellular N-terminus is essential for binding and signal sensing of its cognate ligands, including IL-8, GRO-α (*a.k.a*. CXCL1), etc^9,36,37^. Site-directed mutagenesis demonstrated that acidic residues, E7, D9, E12 and D13, constituted a putative binding pocket for IL-8^38^. Each of the three extracellular loops of *h*CXCR2 was implicated in binding of IL-8 in different studies^39–41^. However, nuclear magnetic resonance and crystallography analyses of the IL-8-*h*CXCR complexes indicated IL-8 binds the N-terminus and loop 2 of the extracellular domain^42–47^. The N-terminus of *h*CXCR1 and *h*CXCR2 share only 25% homology (Fig. 1a**)**, as compared to 100%, 55%, and 92% for loops 1, 2, and 3, respectively, indicating that the extracellular N-terminal peptide of *h*CXCR2 (48 amino acids) could serve as an ideal antigen in panning and optimization of highly selective and potent combinatorial antibodies. Peptide, pepN48 (see Methods and Fig. 1a) was synthesized (Chinese Peptide, Hangzhou) and used in three rounds of phage panning using a 10^11^-member single-chain variable fragment (scFv) combinatorial antibody library^11^. Nine scFv sequences showing high enrichment in the panning were sub-cloned into a *p*Fuse expression vector and their expression and affinity confirmed by ELISA screening of the cellular supernatants with pepN48 (Supplementary Fig. 1a). Further analyses of the supernatants using fluorescence-activated cell sorting (FACS) showed that only one clone, H8 (abN48), recognized the membrane-bound *h*CXCR2-mCherry on U2OS cells (Supplementary Fig. 1b). The H8 (abN48) scFv antibody was overexpressed, purified to homogeneity, and surface plasmon resonance (SPR) analysis indicated its K_D_ value of abN48 with pepN48 was 2.6 × 10^−9^ M (Supplementary Fig. 1c).

### Generation of full-length IgG1 combinatorial antibody and affinity maturation

To optimize binding of abN48, the scFv was first converted into a full-length IgG1 antibody, abN48-IgG1 (Supplementary Fig. 2a) and its K_D_ value with pepN48 was determined to be 7.8 × 10^−10^ M (Fig. 1b). The improved affinity of the IgG1 compared to the scFv suggested the Fab form could be further optimized using affinity maturation by yeast display. To maximize the chance of finding optimal binder(s), all eleven residues of complementarity determining region of heavy chain (H-CDR3), GYCSSTSCYDY, on abN48-IgG1, were randomly mutated. The resulting Fab sequences were displayed on the surface of yeast cells using a *p*ESC vector containing a GAL1-10 bi-directional promoter that expresses both heavy and light chains to generate a combinatorial antibody library of >10^7^ in size. After four rounds of panning against pepN48, 8 highly enriched sequences, abN48-1 to abN48-8, were selected from 100 positive clones and confirmed by DNA sequencing. WebLogo analysis revealed that the most frequent mutations are Ser99 to Arg99 and Ser100A to Arg100A, which was present in abN48-2 (Supplementary Fig. 2b). The 8 Fab antibody sequences were sub-cloned into the *p*Fuse vector and expressed in HEK293T cells. Binding affinity and homogeneity were determined by SPR and size-exclusion and high-performance liquid chromatography (SEC-HPLC), respectively (Supplementary Table 1 and Supplementary Fig. 3). All new constructs showed sub-nanomolar to picomolar binding with pepN48: the K_D_ for abN48-2-IgG1 (Fig. 1b) was 7.2 × 10^−12^ M, which was 100-fold improved over abN48-IgG1. After incubation at 42 °C for 5 days, abN48-2-IgG1 also showed high thermostability with no detectable aggregation or degradation (Supplementary Fig. 3, top row right).

### Epitopes of abN48-IgG1 and abN48-2-IgG1 overlap with IL-8 binding site

We then utilized hydrogen-deuterium-exchange mass spectrometry (HDX-MS) to map the epitopes of abN48-IgG1 and abN48-2-IgG1 on *h*CXCR2. In the presence of abN48-IgG1, the HDX rate of residues 11–18, particularly residues 13–16, on pepN48 decreased considerably (Fig. 1c and Supplementary Fig. 4), indicating their possible location at the interface between abN48-IgG1 and pepN48, in accord with the reported binding of IL-8 to the N-terminus of *h*CXCR2. The major epitope of abN48-2-IgG1 was also identified as residues 13–16 (Fig. 1c and Supplementary Fig. 4). Apparently, the affinity-matured abN48-2-IgG1 preserved the binding mode between abN48–IgG1 and *h*CXCR2, albeit with higher affinity. The HDX protection by both antibodies gradually decreased at longer labeling time points and disappeared at 120 s (Fig. 1c). Considering the negligible off-rate of the antibodies using Biacore (Fig. 1b), these results suggest that side-chain rather than main-chain interactions rendered on peptide form the major stabilization interactions between peptide and antibody.

To identify a minimal interacting peptide sequence of pepN48 for crystallography studies, a series of truncates of pepN48 were constructed. Consistent with HDX-MS mapping, a 9–19 amino acid (aa) peptide (pepN9–19) bound both abN48 and abN48-2 comparable to pepN48, whereas pepN48 lacking residues 9–19 (pepN48Δ9–19) was no longer recognized (Supplementary Fig. 5). Co-crystal structures of the Fabs of both antibodies with pepN9–19 were then determined. The abN48-2 Fab/pepN9–19 complex was refined to 2.2 Å resolution (Supplementary Table 2) with two protein complexes (protomers) per asymmetric unit (Supplementary Fig. 6a). Electron density for residues 11−16 of pepN9-19 could be clearly visualized in both protomers (Supplementary Fig. 6b). Phe14 and Trp15 insert their bulky aromatic side chains into a binding site cavity (Fig. 1d). The benzene ring of Phe14 forms face-to-edge π-π stacking with Phe46^L^ and Tyr34^L^ from abN48-2, and the Trp15 indole is anchored by the side chains of Trp96^L^ and Ser35^H^ (Fig. 1e). Gly95^H^ hydrogen bonds with Phe14 amide nitrogen, while Tyr34^L^ and Trp96^L^ side-chains hydrogen bond with Phe14 and Trp15 carbonyl oxygens (Fig. 1e). The snug fit of Phe14 and Trp15 in the antibody cavity also facilitates hydrogen-bonding from the Ala33^H^ backbone nitrogen to OD2 atom of Asp13, whose OD1 hydrogen bonds with the Trp15 amide nitrogen to further stabilize the peptide conformation (Fig. 1e). The *h*CXCR2 side chains, Phe11, Glu12, and Lys16, also make intimate interactions with abN48-2 Fab (Fig. 1d). Specifically, the Phe11 benzene ring forms face-to-edge π-π stacking with Tyr96^H^, Glu12 salt bridges with Arg32^L^ and Arg49^L^, and Lys16 hydrogen bonds with Asn52^H^ and packs its aliphatic region against Trp50^H^ (Fig. 1e). The Glu12 backbone nitrogen and oxygen also hydrogen bond to the backbone oxygen and nitrogen of Cys97^H^, further strengthening the antibody–antigen interactions (Fig. 1E).

For further epitope validation, we carried out alanine-scan mutagenesis on residues in the N-terminus of *h*CXCR2. Consistent with Phe14 and Trp15 contributing more than 50% (395 Å^2^) of the total (~782 Å^2^) buried solvent-accessible area (SAA) on pepN9-19 upon abN48-2 binding, their mutation completely abolished the interaction between abN48-IgG1 and U2OS cell expressing *h*CXCR2-mCherry fusion protein (Supplementary Fig. 7). Asp13 mutation also had a detrimental effect, while Lys16 mutation did not (Supplementary Fig. 7). Thus, Asp13, Phe14, and Trp15 are the major contributors to the epitope of abN48-2-IgG1 on *h*CXCR2, while Phe11, Glu12, and Lys16 play more auxiliary roles.

As previously proposed^42^, Trp10 from *h*CXCR1 (equivalent to Trp15 of *h*CXCR2), is important for membrane anchoring of the CXCR1 N-terminus. Displacement of Trp10 from the membrane appeared to be a pre-requisite for the subsequent IL-8 binding. Residues 7, 9, 12, and 13 of *h*CXCR2 were implicated in IL-8 binding^38^. Hence, the critical epitope residues of abN48 and abN48-2 identified here, i.e., Asp13-Trp15, overlap with the IL-8 binding site, and, therefore, these antibodies might antagonize *h*CXCR2 by out-competing the natural IL-8 ligand for binding to the *h*CXCR2 N-terminus. AbN48-IgG1 and abN48-2-IgG1 differ by two residues in CDR3, i.e., Ser99^H^ to Arg99^H^ and Ser100A^H^ to Arg100A^H^. Why these arginine substitutions in abN48-2-IgG1 result in a 100-fold increase in binding affinity is not obvious from the x-ray structure and suggests a subtle global stabilization by these residues. Entropy stabilization, thus, could be an important factor in addition to the enthalpy changes in the tight-binding of the optimized antibody, abN48-2-IgG1 with *h*CXCR2.

### Species and sub-type specificity

**Fig. 2:**
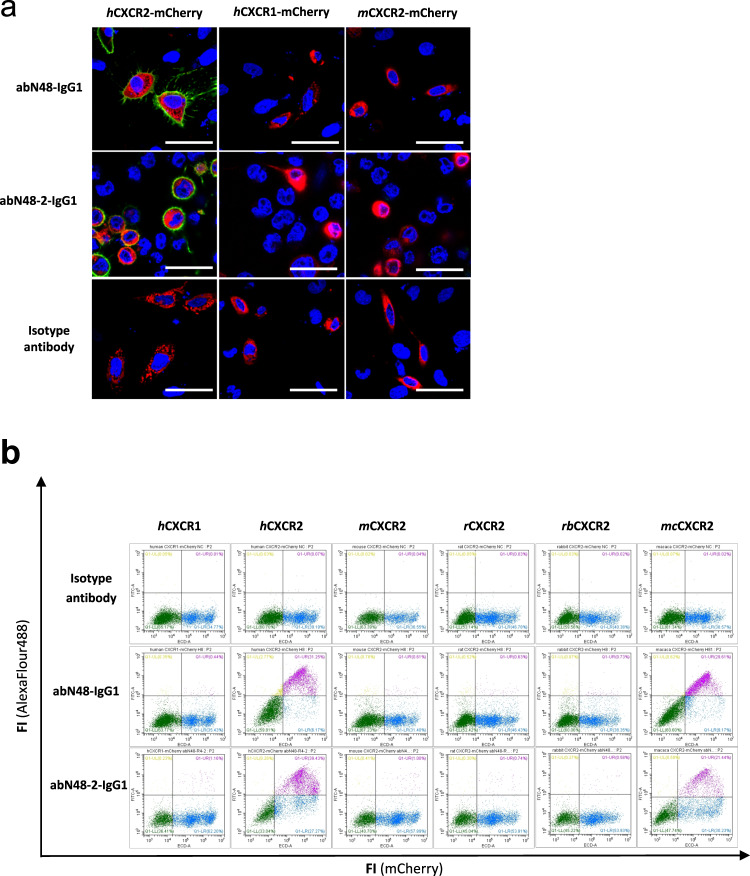
Species and subtype specificity of abN48-IgG1 and ab48-2-IgG1. **a** Representative images of co-localization of abN48-IgG1 or abN48-2-IgG1 to *h*CXCR2, *h*CXCR1, and *m*CXCR2 overexpressed on the membrane of U2OS cells. All test receptor proteins were fused with an mCherry fluorescent tag, and the immunocytofluorescent images were captured by confocal microscopy (3 images of every 3 samples were analyzed for each group and all gave consistent results). Scale bars (white) represent 50 μm. **b** Surface interactions of abN48-IgG1 and abN48-2-IgG1 with *h*CXCR2, *h*CXCR1, *m*CXCR2, *r*CXCR2, *rb*CXCR2, and *mc*CXCR2 overexpressed on U2OS cells. Flow-cytometry was used to measure the interactions between antibodies and the CXCR2 receptor proteins. Isotype-antibody (irrelevant human IgG1 antibody) was used as a negative control. For each sample, 3 repeats were carried out and all gave consistent results.

To determine the species and sub-type specificities of abN48-IgG1 and abN48-2-IgG1, gene constructs containing *h*CXCR1-mCherry, *h*CXCR2-mCherry, mouse CXCR2 (*m*CXCR2-mCherry), rat CXCR2 (*r*CXCR2-mCherry), rabbit CXCR2 (*rb*CXCR2-mCherry), and macaque CXCR2 (*mc*CXCR2-mCherry) were overexpressed in U2OS cells. Immunofluorescence co-localization of abN48-IgG1 and abN48-2-IgG1 on the surface of U2OS cells overexpressing *h*CXCR1-mCherry, *h*CXCR2-mCherry, *m*CXCR2-mCherry showed both antibodies specifically recognize surface *h*CXCR2 (Fig. 2a). This specificity was further confirmed by FACS analyses, in which both antibodies bound exclusively to *h*CXCR2 and closely related macaque monkey CXCR2 (72.9% identity), but not to CXCR2 of mouse (45.8% identity), rat (43.8% identity), rabbit (*58.3%* identity), or to *h*CXCR1 (Fig. 2b).

### Potent inhibition of *h*CXCR2-mediated β-arrestin signaling and calcium influx

To understand cellular functions of the *h*CXCR2-specific antibody binders, two independent signaling pathways mediated by CXCR2, namely β-arrestin recruitment and cytoplasmic Ca^2+^ influx, were evaluated using Tango and FLIPR methods, respectively. β-arrestin recruitment is a G protein-independent intra-cellular event; whereas Ca^2+^ influx is a result of its release from the endoplasmic reticulum (ER) into the cytoplasm in response to IP3 produced by Gα protein activation. Both events are known to be directly associated with CXCR2 activation.

**Fig. 3:**
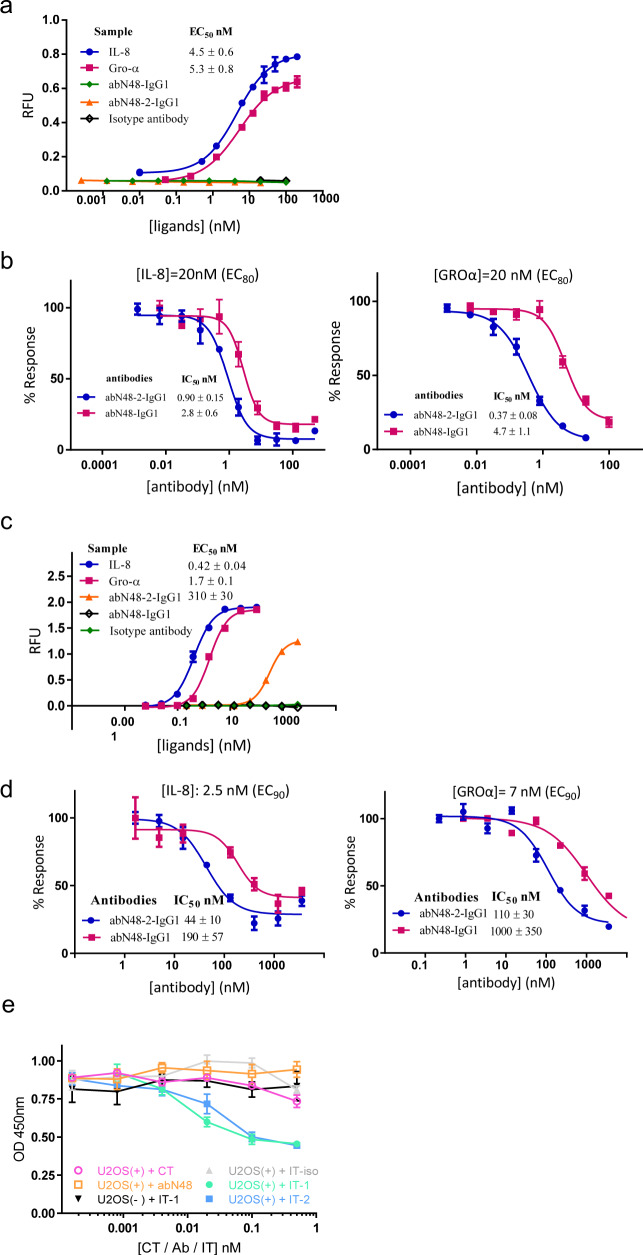
CXCR2 mediated cellular signaling pathways. **a** Activation of β-arrestin signaling by IL-8, GRO-α, and the antibody ligands using a Tango reporter gene assay. **b** Inhibition of chemokine-induced β-arrestin signaling by abN48-IgG1 and abN48-2-IgG1. The induction concentration of IL-8 (left) and GRO-α (right) were at their corresponding EC_80_ values. **c** Activation of Ca^2+^ influx by IL-8, GRO-α, and the abN48 antibody ligands using FLIPR measurement. **d** Inhibition of chemokine-induced Ca^2+^ influx by abN48-IgG1 and abN48-2-IgG1. The induction concentration of IL-8 (left) and GRO-α (right) were at their corresponding EC_90_ values. Data are represented as mean ± standard deviation. The calculated EC_50_ and IC_50_ values are listed next to the fitted curves. (For Tango and Ca^2+^ assay (**a**–**d**), *n* = 3 independent reporter cell samples measured in a single experiment, data presented as mean (center) ± s.d. (error bars). Each assay was repeated 3 times and all gave consistent results). **e** Antibody-dependent CXCR2 internalization. U2OS( + ) and U2OS(−) represent U2OS cells with and without surface *h*CXCR2 expression, respectively; CT represents the cytotoxin AL1-PE38KDEL; abN48 represents the antibody without cytotoxin; IT-iso, IT-1, and IT-2 represent the immunotoxins (ITs) assembled with an irrelevant human IgG1 antibody, abN48-IgG1 and abN48-2-IgG1 to the cytotoxin AL1-PE38KDEL, respectively. (*n* = 3 independent cell samples tested in a single experiment, data presented as mean (center) ± s.d. (error bars). This assay was repeated twice and gave consistent results.).

Using Tango assays, two natural ligands and the two selected antibodies were tested for agonistic effects in β-arrestin recruitment. Only natural ligands, IL-8 and GRO-α, induced β-arrestin recruitment, with apparent 50% effective concentration (EC_50_) of 4.5 and 5.3 nM, respectively (Fig. 3a), whereas abN48-IgG1 and abN48-2-IgG1 showed no activation up to 100 nM. However, the antibodies showed complete inhibition of natural ligand-induced recruitment of β*-*arrestin (at EC_80_ level), with apparent 50% inhibitory concentration (IC_50_) of 2.8 and 0.90 nM for IL-8, and 4.7 and 0.37 nM for GRO-α, respectively (Fig. 3b). Both antibodies also showed an inverse agonist effect, in which antibody at 20 nM markedly suppressed the intrinsic β-arrestin recruitment by CXCR2 over-expression (Supplementary Fig. 8).

The natural ligands and antibodies were then tested for their ability to stimulate Ca^2+^ influx. IL-8 and GRO-α showed dose-dependent activation with EC_50_ values of 0.42 and 1.7 nM, respectively (Fig. 3c), whereas only the tighter binding antibody, abN48-2-IgG1, displayed a partial agonist effect at high concentration (10–1000 nM), with apparent EC_50_ of 310 nM (Fig. 3c). However, both antibodies displayed dose-dependent inhibition of natural ligand-induced Ca^2+^ influx (at EC_90_ level), with IC_50_ values of 190 and 44 nM, respectively for IL-8, and 1000 and 110 nM, respectively for GRO-α (Fig. 3d). The observed enhancement on CXCR2-mediated signaling by abN48-2-IgG1 is consistent with its improved binding to the CXCR2 N-terminus.

### Potent induction of endocytosis of *h*CXCR2

IL-8 also induces endocytosis signaling of CXCR2 at 5–10 nM^48^. To test any antibody effect, internalization of CXCR2 was studied using U2OS cells overexpressing membrane-bound *h*CXCR2. Immunotoxin complexes of abN48-IgG1 or abN48-2-IgG1 with a cytotoxin AL1-PE38KDEL were used as molecular probes. The immunotoxin complex of abN48-IgG1 or abN48-2-IgG1 showed dose-dependent cytotoxicity of U2OS cells overexpressing *h*CXCR2 (Fig. 3e), whereas U2OS cells without *h*CXCR2 expression, and an immunotoxin complex of an irrelevant antibody, showed no detectable cytotoxicity. Both abN48-IgG1 and abN48-2-IgG1 showed extremely potent activation of CXCR2 internalization with estimated EC_50_ of less than 0.1 nM.

### Potent inhibition of IL-8-induced neutrophil chemotaxis

**Fig. 4:**
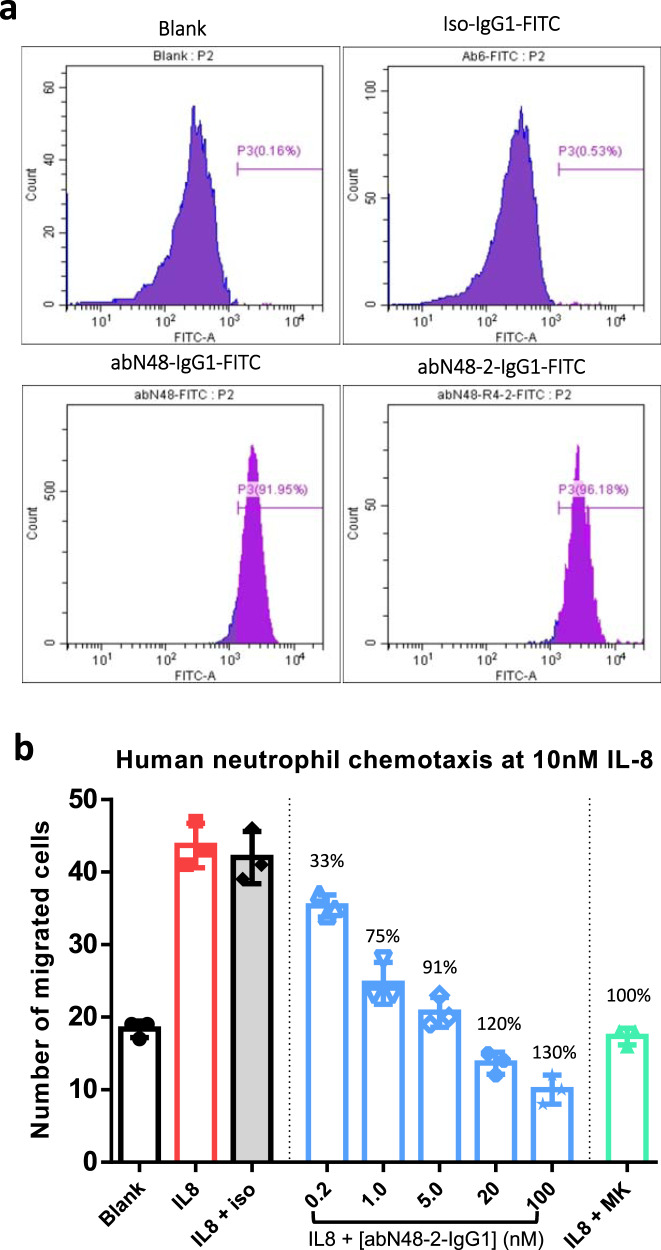
Inhibition of IL-8 induced neutrophil chemotaxis. **a** Representative flow-cytometry results showing abN48-IgG1 and abN48-2-IgG1 (FITC-conjugated) specifically bind to human primary neutrophils. Blank: cells without *h*CXCR2 expression; iso-IgG1-FITC: irrelevant human IgG1 antibody conjugated with FITC. (3 independent biological replicates showed the same results) **b** Inhibition of abN48-2-IgG1 on IL-8 (10 nM) induced neutrophil chemotaxis was determined by a chemotaxis assay. Blank: media without IL-8 or inhibitor; IL-8: 10 nM IL-8; iso: 10 nM irrelevant human IgG1 antibody; MK: 1 μM small molecule CXCR2 inhibitor MK7123. The values of percentage (%) above the blue and green bars represent the percentage of inhibition of neutrophil chemotaxis induced by IL-8 at various concentrations of abN48-2-IgG1 and 1 μM MK7123, respectively. (*n* = 3 independent cell samples measured in a single experiment, data presented as dots overlapped with mean (column) ± s.d. (error bars). 3 independent experiments with different samples and antibody concentrations showed consistent results).

Neutrophil chemotaxis occurs along the gradient of its corresponding chemokine secreted at a distant acutely damage or infected site^49^. Since CXCR2 is known to be expressed mainly on neutrophils, and its cognate ligand IL-8 is a key regulator in many pathological processes, the IL-8-CXCR2 axis constitutes an important target for therapeutic intervention^50^. We therefore tested abN48-2-IgG1 in a chemotaxis assay using primary human neutrophils. Viable neutrophils were negatively selected via immuno-magnetic bead separation from a whole blood collection, and their purity was verified by immune-staining of CD15^+^, CD16^+^, CD11b^+^, and CD66b^+^ (MT-Bio, Shanghai). We examined membrane expression of *h*CXCR2 on neutrophils using abN48-IgG1 and abN48-2-IgG1 (Fig. 4a). FACS analyses showed that the whole population of neutrophils was captured by our CXCR2-specific antibodies. Next, the effect on IL-8-induced neutrophil chemotaxis was tested in a trans-well migration assay. The abN48-2-IgG1 showed dose-dependent inhibition of neutrophil chemotaxis induced by a maximal IL-8 concentration (10 nM) with 75% inhibition at 1 nM and 120% inhibition at 20 nM **(**Fig. 4b). For comparison, a small molecule CXCR1/CXCR2 dual inhibitor, MK7123, showed 100% inhibition of neutrophil chemotaxis at 1 μM. The observation of complete blockage of neutrophil migration (both chemokine-dependent and chemokine-independent) by abN48-2-IgG1 may suggest the involvement of CXCR2 in the basal intrinsic neutrophil migration.

### Establishment of *h*CXCR2 knock-in mice

Since abN48-2-IgG1 showed an exclusive species specificity (Fig. 2), a homozygous strain of *h*CXCR2 knock-in mice (*h*CXCR2 mouse) was first generated by replacement of the *cxcr2* CDS in the genome of C57BL/6 mice with that of human via gene targeting in the corresponding embryonic stem cells. Both the genotype and phenotype of *h*CXCR2 knock-in were validated by sequencing and flow-cytometry. As expected, abN48-2-IgG1 was only able to stain the neutrophils from *h*CXCR2 knock-in not wild-type mice (Supplementary Fig. 9). The abundance of circulating neutrophils appeared to not be affected by *h*CXCR2 knock-in (Supplementary Fig. 9). Since mice do not secrete IL-8 or CXCL6, *m*CXCL1, the murine orthologue of human chemokine Gro-α, was tested for its chemokine activity on *h*CXCR2 mediated calcium signaling. Comparing to human Gro-α (Fig. 3c), *m*CXCL1 showed a compatible stimulation of calcium influx with a similar EC_50_ value (1.7 nM *vs*. 5.6 nM, respectively) (Supplementary Fig. 10a). Furthermore, abN48-2-IgG1 completely inhibited the *m*Cxcl1 induced *h*CXCR2 calcium influx with an almost identical IC_50_ value at EC_90_ concentration of *m*CXCl1 (Supplementary Fig. 10b).

### Efficacy of AbN4-2-IgG1 and SB225002 in *h*CXCR2 mediated EAE

**Fig. 5:**
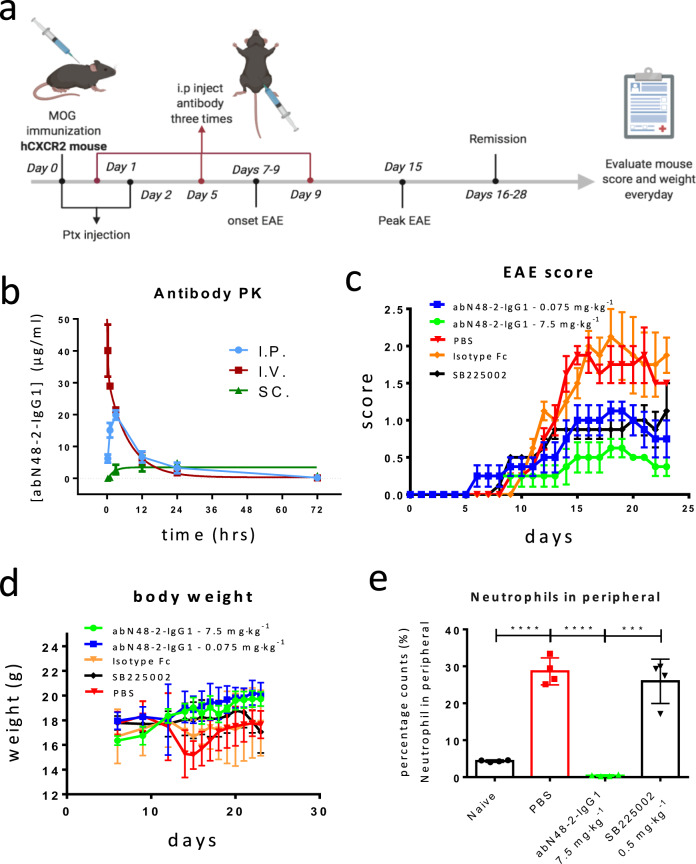
Alleviation of EAE symptoms in *h*CXCR2 knock-in mice by abN48-2-IgG1. **a** Schematic illustration of abN48-2-IgG1 treatment in an EAE mouse model. **b** Result of PK study of abN48-2-IgG1 in *h*CXCR2 knock-in mice (*n* = 4 mice per group). **c**, **d** EAE score and body weight of *h*CXCR2 knock-in mice during abN48-2-IgG1 treatment (*n* = 4 mice per group). **e** Comparison of neutrophils in peripheral blood of experimental mice. One blood sample was collected from each mouse in the indicated groups: “Naive” represents the group (*n* = 4 mice) without EAE stimulation; “isotype Fc” represents the group (*n* = 4 mice) treated with negative control (Fc); “PBS” represents the group (*n* = 4 mice) with EAE stimulation and PBS treatment; and “abN48-2-IgG1” represents the group (*n* = 4 mice) with EAE stimulation and abN48-2-IgG1 treatment (7.5 mg kg^−1^). “SB225002” represents the group (*n* = 4 mice) with EAE stimulation and daily treatment with CXCR2-selective inhibitor SB225002 (0.5 mg kg^-1^). For panel **b** to **d**, data are presented as mean (center) ± s.d. (error bars). For panel **e**, data are presented as dots overlapped with mean (column) ± s.d. (error bars). Two-tailed t-test was employed for the significance of differences. *p* < 0.0001 (*p* < 0.0001, ****) for t-test of “PBS” *vs*. “Naive” and “abN48-2-IgG1” *vs*. “PBS”, *p* = 0.0001 (*p* < 0.001, ***) for t-test of “abN48-2-IgG1” *vs*. “SB225002”. t-values and degree of freedom were referred to the “Methods” section.

Activation and migration of neutrophils are known to play a key role in the rodent experimental autoimmune encephalomyelitis (EAE) model^51–53^, which mimics the autoimmune component of human multiple sclerosis. Using the above *h*CXCR2 knock-in mice, we established an EAE model and tested the efficacy of *h*CXCR2 specific antibody, abN48-2-IgG1. The pharmacokinetics of abN48-2-IgG1 in *h*CXCR2 mice were first examined (Fig. 5b). The naïve *h*CXCR2 mice were dosed individually with abN48-2-IgG1 via intraperitoneal (*i.p*.), subcutaneous (*s.c*.), or intravenous (*i.v*.) injections. Time courses of plasma concentration changes of abN48-2-IgG1 were monitored for 72 h. The antibody showed an excellent half-life of t_1/2_ > 11 h and bioavailabilities of 73% and 95% for *s.c*. and *i.p*. dosing, respectively (Supplementary Table 3).

The dosing schedule for the establishment of EAE in *h*CXCR2 knock-in mice and the following antibody treatment is illustrated in Fig. 5a. Two different doses of abN48-2-IgG1 (7.5 mg kg^−1^ and 0.075 mg kg^−1^) were studied via *i.p*. injection (4 mice per group). Vehicle (PBS) and negative (isotype Fc) controls were administrated according to the same dosing schedule as that for the antibody. SB225002, a selective small molecule inhibitor against *h*CXCR2 (150-folds *vs. h*CXCR1), was used as a benchmark. SB225002 was *i.p*. dosed *qd* at its maximum efficacious concentration (0.5 mg kg^−1^^52,54,55^) over the course of EAE development. Results showed that comparing to negative and vehicle controls, abN48-2-IgG1 alleviated the EAE symptoms of *h*CXCR2 knock-in mice in a dose-dependent manner (Fig. 5c). At 7.5 mg kg^−1^, abN48-2-IgG1 attenuate about 90% EAE scores comparing to about 60% attenuation observed for the benchmark SB225002. It was also noted in a separate experiment that abN48-2-IgG1 did not have any effect on the EAE symptoms in the wild type mice. Furthermore, the plasma neutrophils at 24 h after the first injection of abN48-2-IgG1 were measured and showed a nearly complete attenuation even comparing to the basal plasma neutrophils of naive *h*CXCR2 mice (without myelin oligodendrocyte glycoprotein peptide or pertussis toxin (PTX) challenge) (Fig. 5e).

## Discussion

Biased signaling is a general feature for GPCRs because of the special structure and function of these receptors in cells. The extracellular domains, including the N-terminus and loops of the seven transmembrane regions, provide a combination of diverse sequences capable of sensing differential stimulation from ligands of various origins. Thus, different ligands that sense the same combination of loops or epitopes on the receptor may be expected to have similar cellular functions. This notion appears to be supported in our study of the CXCR2 signaling network with IL-8 and GRO-α. However, in many physiological and pathological situations, structurally different ligands acting on the same receptor are able to preferentially activate only one signaling pathway, which is a phenomenon termed biased agonism or functional selectivity that has been studied extensively, especially for chemokines and chemokine receptors^56,57^. The combinatorial antibody library technology with its vast numbers of diverse protein binders can therefore be an invaluable tool in decoding the epitope combinations that are essential for biased signaling of membrane receptors.

Our findings here show that, through epitope-guided combinatorial enrichment, monoclonal antibodies can be identified that can directly compete with IL-8 for binding to its cognate epitope on *h*CXCR2, and are substantially enhanced in potency and efficacy over previous attempts using proteoliposomes or live cells formats that display full length on *h*CXCR2 surface, chimeric antigen or biparatopic nanobody format^7,8^. The optimized antibody, abN48-2-IgG1, displayed a biased agonism profile when tightly bound to CXCR2 N-terminus, a partial agonistic effect on G-protein activation-associated Ca^2+^ influx at high concentration, an inverse agonistic effect on β-arrestin recruitment, and an extremely potent stimulatory effect on CXCR2 endocytosis. For IL-8 and GROα, the N-terminal epitope of CXCR2 appears to constitute an exclusive β-arrestin signaling response for these natural ligands. However, tight binding of the combinatorial antibody to the N-terminus of CXCR2 did not abolish G-protein activation in cells, but greatly enhanced receptor internalization. This combination of complete blockage of β-arrestin recruitment and highly sensitized CXCR2 endocytosis was synergistic to inhibition of neutrophil migration. Low nM concentrations of abN48-2-IgG1 completely abolished induction of human neutrophil chemotaxis by 10 nM IL-8. Considering that elevated levels of IL-8 in patient serum is in the picomolar to sub-nanomolar range^58–60^, abN48-2-IgG1, with a defined mechanism of inhibition, shows great potential for clinical development. The N-terminal epitope of *h*CXCR2 for abN48-2-IgG1 ligand (pepN11-16) thus is most likely a biased cognate site for mitogenic signalings of CXCR2. Considering the facts that between CXCR1 and CXCR2, the N-terminus is the least conserved extracellular region amongst the other three extracellular loops, and CXCR2 is known to have at least 7 cognate ligands, compared to only 2 ligands for CXCR1, the N-terminus of CXCR2 most likely plays a key role in ligand biased agonisms.

In the animal study, the tight-binding antibody, abN48-2-IgG1, showed an exclusive dose-dependent anti-EAE efficacy in the *h*CXCR2 knock-in mice, which appeared to correlate with its effective suppression of plasma neutrophils at the onset of EAE. Compared to a small molecule allosteric CXCR2 selective inhibitor, the antibody demonstrated more superior efficacy. The results of two independent experiments for abN48-2-IgG1, a nearly complete loss of neutrophils in the blood of experimental mice as compared to naïve mice controls mice (Fig. 5e), and a robust further inhibition (30%) of basal neutrophil migration in the Boyden chamber (Fig. 4b), are striking since no small molecule inhibitors with similar effects have been reported. The tight-binding character of abN48-2-IgG1 appears to play an essential role. Our current study demonstrates the great potential of abN48-2-IgG1 in the treatment of various autoimmune-based, complex chronic diseases including MS, rheumatoid arthritis, and COPD, etc. In addition, elucidating the precise combination of loops and epitopes used by natural GPCR ligands would further our understanding of the signaling networks and interplay among different receptors and cell types.

## Methods

### Nomenclature

Amino acid sequences are represented in single-letter or three-letter codes and the sequences of antibody are shown in Kabat numbering.

### Cell culture

HEK293T cells (#GNHu17; cell bank of Chinese Academy of Science, Shanghai) were maintained in DMEM medium (#12800017; Gibco) containing 10% (v/v) FBS (#100-500; Gemini). HEK293F cells (#R79007; Gibco) were cultured in FreeStyle 293 Expression Medium (#12338-026; Gibco). U2OS cell line (#SCSP-5030; cell bank of Chinese Academy of Science, Shanghai) was maintained in McCoy’s 5 A Medium (#16600-082, Gibco) containing 10% (v/v) FBS. Tango^TM^ CXCR2-bla U2OS cell line (#K1807; Thermo Fisher Scientific) according to the manufacturer’s instructions. CHO-K1 cells were cultured in Ham’s F-12K medium (#21127022; Gibco) containing 10% (v/v) FBS. Cell lines were authenticated upon arrival by STR profiling and tested for mycoplasma contamination periodically.

### Combinatorial antibody library panning

A peptide consisting of the first 48 residues of the extracellular N-terminus of *h*CXCR2 (MEDFNMESDSFEDFWKGEDLSNYSYSSTLPPFLLDAAPCEPESLEIN K) with biotin labeled on its N-terminus was synthesized (Chinese Peptide), and used as the antigen (pepN48) for phage panning. The panning procedure followed a modified protocol as described previously^61^. Briefly, phage particles displaying a combinatorial scFv antibody library with ~10^11^ diversity were incubated with pepN48 antigen. Streptavidin-coated magnetic beads (1:1000 dilution; #21925, Pierce) were then added to the solution to pull down the phage-bound biotinylated antigen. Bound phages were eluted with glycine-HCl (pH 2.0) after 3 washes to remove unbound phages, and used in the following infection of XL-1 blue cells (#200228; Agilent). The infected cells were used to generate phage particles for next round of panning in the presence of helper phage VCSM13 (#200251; Agilent). After 3 rounds of enrichment, colonies were picked and tested by phage ELISA, after which all positive clones were DNA sequenced. The international ImMunoGeneTics information system (IMGT) was used for contig analysis of sequences of these colonies. Nine distinct scFv sequences were found to be highly enriched.

### ELISA

Avidin (#21121; Pierce) was diluted in carbonate-bicarbonate buffer pH 9.6 (#C3041; Sigma) to a final concentration of 2 ng μL^−1^. 96-well ELISA plates (Corning Costar) were coated by incubation with avidin solution (25 μL per well) at 4 °C overnight. Wells were washed once with 150 μL per well PBST buffer (0.05% TWEEN20 in PBS pH 7.4). A total of 50 ng of pepN48 in PBS pH 7.4 (2 ng μL^−1^) was added to each well, and incubated for 30 min at room temperature. Wells were washed 3 times with PBST buffer, and blocked with M-PBST (3% milk in PBST, 150 μL per well) at 37 °C for 5 min. After removal of M-PBST, 25 μL sample solution containing purified antibody or phage supernatant (diluted in M-PBST to proper concentration) were added into each well, and incubated for 1 h at room temperature, followed by 5 washes with PBST. Anti-M13 HRP-conjugated secondary antibody (1:3,000 dilution; #27-9421-01, GE) or anti-human Fc HRP-conjugated secondary antibody (1:3,000 dilution; #A0170, Sigma) was added into wells, incubated for 1 h at room temperature, washed five times with PBST, and followed by incubation with 50 μL per well ABTS solution (#11684302001; Roche) at room temperature for 20 min. Absorbance at 405 nm was measured on a plate reader (EnSpire; PerkinElmer).

For abN48-2-IgG1 animal PK study, pepN48 in PBS was coated on 96-well plates at 0.1 μg per well. HRP-labeled anti-human IgG antibody (1:1000 dilution; Promega, #W403B) was used for detection. Other reagents and work flow of ELISA were the same as described above. Sample preparation is described in PK study section in the “Methods”.

### Expression and purification of antibodies

DNA sequences encoding the candidate scFv antibodies were cloned into a *p*Fuse expression vector (#pfuse-hg1fc2; InvivoGen) for expression of scFv-Fc proteins with the entire Fc domain of human IgG1. For antibodies in the full-length IgG1 format, variable regions of heavy chain and light chain (V_H_ and V_L_) from the scFv sequence were cloned into plasmids with the complete constant domains of IgG1 heavy chain and light chains (C_H_ and C_L_). The antibodies were expressed through transfection of the scFv-Fc expression plasmid, or co-transfection of equal molar of heavy chain and light plasmids for full-length antibody, into HEK293F cells followed by cell culture for 5 days. Antibodies in the medium were purified with a HiTrap Protein A HP column (#17-0403-03; GE Healthcare) by ÄKTAxpress purifier (GE Healthcare). Purified antibodies were concentrated and stored in PBS buffer (pH 7.4) at −80 °C.

### SEC-HPLC

SEC-HPLC was carried out to determine the thermostability and homogeneity of purified recombinant antibodies. Briefly, antibody solutions were first concentrated to 20 mg·mL^−1^, and incubated at 42 °C for 5 days. The resulting antibody solutions were analyzed by HPLC on an SEC column (Nanofilm SEC-250) with a running buffer of 0.05% DDM / 0.01% CHS in Tris (pH 8.1). Aggregation and degradation of the combinatorial antibody over prolonged incubation at 42 °C were evaluated and compared by homogeneity of its corresponding elution peak.

### Flow-cytometry

The interaction of antibody ligand with surface chemokine receptors was studied using cell lines (U2OS or HEK293T) transiently overexpressing the corresponding chemokine receptor. After 24 h, cells were detached from dish and re-suspended in a FACS buffer (0.5% BSA in PBS pH 7.4, with 2 mM EDTA) to 5 × 10^6^ cells per mL. 500,000 cells per tube were incubated with 2 μg mL^−1^ corresponding antibody in a FACS buffer at 4 °C for 15 min. The resulting cells were washed twice with a FACS buffer, and then incubated with 2 μg mL^−1^ secondary antibody, Alexa Fluor^TM^ 488 goat anti-human IgG (H + L) (1:1000; #A11013, Invitrogen), at 4 °C for 15 min. After two washes with the FACS buffer, cells were re-suspended in PBS and analyzed by CytoFLEX S (Beckman Coulter).

For analysis of neutrophils in the animal study, peripheral blood was isolated from canthus vein of mice. After lysis of red blood cells using RBC lysing solution (#420301, Biolegend), whole blood cells were washed twice with PBS and stained for 30 min at 4 °C with the following antibodies: CD11b Monoclonal Antibody (M1/70.15), PE (1:1000 dilution; #RM2804, Invitrogen); Ly-6G/Ly-6C Monoclonal Antibody (RB6-8C5), FITC (1:1000 dilution; #11-5931-82, eBioscience); anti-mouse CD182 Antibody (REA942), APC (1:1000 dilution; #130-115-635, Miltenyi); anti-human CD182 antibody (REA208), FITC (1:1000 dilution; #130-104-886, Miltenyi); abN48-2-IgG1 and Alexa Fluor^TM^ 633 goat anti-human IgG (H + L) (#A-21091; Invitrogen). Sample data were acquired on CytoFLEX (Beckman) following wash with and re-suspended in PBS containing 0.5% BSA.

### Immunofluorescence assay

Cells were plated and cultured in poly-D-lysine-coated, glass-bottom, 96-well microtiter plates (ViewPlate microplate (#6005530, PerkinElmer). Plasmids expressing target membrane proteins were transfected into cells using Lipofectamine 3000 (#L3000015; Invitrogen). 24 h after transfection, cells were fixed by 4% paraformaldehyde for 20 min at room temperature, followed by 3 washes with PBS (pH 7.4) for 5 min each. Cells were then blocked with 1% BSA (#V900933, Sigma; dissolved in PBS) for 30 min at 37 °C. After blocking, cells were incubated with corresponding antibodies (diluted in 1% BSA/PBS, 2 μg mL^−1^) at 4 °C overnight. 2 μg mL^−1^ secondary antibody, Alexa FluorTM 488 goat anti-human IgG (H + L) (1:1000 dilution; #A11013, Invitrogen), was then mixed with the cells in a 1% BSA/PBS buffer at room temperature for 1 h. DAPI (#10236276001, Roche) was added for nuclei staining. After 3 times washing with PBS, the stained cells were kept in PBS for immunofluorescence analysis on a confocal microscopy (ZEISS LSM710).

### Surface-plasmon-resonance

Biotinylated antigen (purified protein or peptide) was loaded on the streptavidin coated surface of a Series S Sensor Chip SA (#BR100531; GE healthcare) to form a single molecular layer. The binding interactions between antibody and antigen were measured on a Biacore T200 system (GE Healthcare). Briefly, testing antibodies were serially diluted in a running buffer, HBS-EP + buffer pH 7.4 (BR100669; GE Healthcare), to concentrations of 0.5, 1, 2, 5, 10 nM, and flowed through the surface of chip for the detection of binding between antigen and antibody. Kinetics of association/dissociation were measured and fitted to an appropriate protein-protein interaction model to calculate the corresponding binding constant (K_D_).

### Affinity maturation using yeast display

DNA sequences of the heavy and light chains of the selected antibody (abN48-IgG1) were cloned into a yeast surface display vector *p*ESC-HA. Primers containing a randomly mutated H-CDR3 region were used to generate CDR3-diversified heavy chains using overlap PCR. The CDR3-diversified heavy chain fragments and linearized backbone plasmid were transfected into EBY100 yeast cells by electroporation to complete construction of the yeast-based Fab mutagenic library using the intrinsic homologous recombination strategy of yeast cells.

The antibody display and analysis by flow-cytometry was performed as described previously^62^. Briefly, EBY100 yeast cells transformed with the mutagenic library were grown in SD/Trp^-^ media (#630308; Clontech) to OD_600_ = 1 and then induced in SG/R-CAA medium at 20 °C for 18–24 h with shaking for expression induction of Fab antibody fused with a c-myc tag on the yeast surface. After the cells were washed with FACS buffer (0.5% BSA in PBS with 2 mM EDTA), pepN48 and anti-c-myc chicken IgY polyclonal antibody (1:500 dilution; #A21281, Invitrogen) were added to the cell suspension and incubated for 30 min at room temperature. Cells were washed 3 times with FACS buffer, and R-phycoerythrin-conjugated Streptavidin (1:1000 dilution; #21627, Invitrogen) and FITC-conjugated goat anti-chicken secondary antibody (1:500 dilution; #PA1-28794; Invitrogen) were then added to the cell suspension and incubated at 4 °C for 30 min. Finally, cells were analyzed by a flow cytometer (CytoFLEX S; Beckman Coulter) after being washed 3 times with FACS buffer. For the library screen, fluorescence-labeled yeast cells of each round were sorted with a flow cytometer (FACSAria III; BD). Concentration of pepN48 was decreased gradually in each round of selection from 1 nM to 0.25 nM.

### Tango assay

In the Tango assay, *h*CXCR2 was fused to an exogenous transcription factor, with a specific cleavage sequence for a non-native protease fused with β-arrestin linked in between. Once ligands bind to *h*CXCR2 and trigger desensitization of membrane, the intracellular arrestin-protease fusion protein is recruited to the activated receptor. The fused transcription factor is then cleaved by the protease, enters into the nucleus, and results in activation of a reporter gene converting substrate to product with fluorescence emission at 520 nm and 447 nm wavelength, respectively. β-arrestin used in this study is beta arrestin 2 (ARRB2, NM_004313), linked to a TEV protease site and a Gal4-VP16 transcription factor stably integrated in a commercial beta-lactamase reporter cell line named Tango™ CXCR2-bla U2OS (#K1521, Thermo). The Tango assay was performed following the manufacturer’s instruction of LiveBLAzer FRET-B/G Loading Kit (#K1030; Invitrogen). Briefly, Tango CXCR2-*bla* U2OS cells were plated at 20,000 cells per well on a CellCarrier-96 (#6005550; PerkinElmer) plate and cultured in growth media at 37 °C / 5% CO_2_ overnight. The resulting cells were re-inoculated into the assay medium (FreeStyle™ Expression Medium without supplement; #12338-018, Invitrogen), and incubated for 48 h at 37 °C/5% CO_2_. Different concentrations of ligands, such as IL-8, GRO-α and antibody, were mixed with the above cells at 37 °C/5% CO_2_ overnight to measure their agonist effects.

For inhibition studies of abN48-IgG1 and abN48-2-IgG1, cells were first treated with antibody at serial dilutions (100, 20, 4.0, 0.80, 0.16, 0.032, 0.0064, 0.0013 nM) for 30 min at 37 °C/5% CO_2_, then 20 nM IL-8 or GRO-α (EC_80_) was added and incubated overnight at 37 °C/5% CO_2_. Before lysing the cells, detection substrate mixture was added and incubated with cells for 2 hours at room temperature. The fluorescence emissions at wavelength 520 nm and 447 nm upon excitation at wavelength 409 nm were recorded and quantitated on a fluorescence plate reader (EnVision, PerkinElmer).

### Calcium influx

Calcium ion influx assays were carried out on in-house CHO cells stably expressing both *h*CXCR2 and Gα16 protein. Cells were plated at 20,000 cells per well in a 384-well plate and cultured for 4–6 h in growth media supplemented with 1% FBS. Media was removed and the resulting cells were washed twice with HBSS buffer (#14025092; Gibco). 25 μL of the loading solution containing freshly prepared calcium dye, Fluo-4 Direct^TM^ (#F10471; Invitrogen), were added into each well and incubated for 30 min at 37 °C. The assay plate was allowed to equilibrate to room temperature before subject to FLIPR detection. For measurement of an agonist effect, 5 μL ligand stocks (in HBSS buffer) of different concentrations for IL-8, GRO-α, and combinatorial antibodies, were mixed quickly into each well containing the dye-loaded cells, and recorded immediately on a FLIPR reader (Tetra Multi-Mode Microplate Reader, Molecular Devices) at wavelength 494 nm (excitation) and 516 nm (emission). The final assay concentrations were 100, 25, 6.2, 1.6, 0.39, 0.098, 0.024, 0.0061 nM for IL-8 and GRO-α and 3600, 900, 225, 56, 14, 3.5, 0.88, 0.22 nM for abN48-IgG1and abN48-2-IgG.

For inhibition studies of abN48-IgG1 and abN48-2-IgG1, 5 μL HBSS stocks with different concentrations of abN48-IgG1 and abN48-2-IgG1 were first mixed into each corresponding well containing 25 μL dye-loaded CHO cells, followed by equilibrium at room temperature for 30 min. Ca^2+^ influx signals were initiated by quick mixing of 5 μL HBSS stock of IL-8 (final concentration in the assay of 2.5 nM) or GRO-α (final concentration in the assay of 7 nM) into each well on FLIPR. The final concentrations of abN48-IgG1 and abN48-2-IgG1 in the assay were 3600, 1200, 400, 130, 44, 15, 4.9, 1.6 nM for IL-8 inhibition, and 3600, 900, 225, 56, 14, 3.5, 0.88, 0.22 nM for GRO-α inhibition, respectively. Fluorescent signals of Ca^2+^ influx were recorded in real time as described above.

### Internalization of *h*CXCR2

A modified method utilizing an adaptor-toxin fusion protein, AL1-PE38KDEL, which binds specifically to IgG and forms an immunotoxin complex, was used to measure the antibody-stimulated CXCR2 endocytosis^63^. The resulting immunotoxin complex induces cell death when it enters cells. Briefly, U2OS cells overexpressed with *h*CXCR2 (or without *h*CXCR2 expression as control) were seeded in 96-well plate and cultured at 37 °C/5% CO_2_ overnight. The next day, AL1-PE38KDEL (provided by Prof. Sachdev Sidhu’s lab) was added to antibody solutions at a 1:1 molar ratio and incubated at room temperature for 1 h to generate the immunotoxin complex. Cell medium was changed to Opti-MEM Reduced Serum Medium (#31985070; Gibco). Then antibody only, or cytotoxin only, or assembled immunotoxin, was mixed with cells at different final concentration of 1.6 × 10^−4^, 8.0 × 10^−4^, 4.0 × 10^−3^, 0.020, 0.10, 0.50 nM, and incubated for 4 h at room temperature. The resulting cells were re-inoculated into a fresh media and cultivated for 3 days at 37 °C/5% CO_2_. WST-1 (#W201-12; Dojindo) was used to determine cell viability by measuring the absorbance at 450 nm on a plate reader (EnSpire: PerkinElmer) according to the manufacturer’s protocol.

### Neutrophil chemotaxis

Neutrophil migration was detected with 24-well transwell chambers with 8-μm pore size membranes (#3422, Corning Costar). Primary human neutrophils (#PBN-1F, MT-Bio) were suspended in chemotaxis medium without phenol red (RPMI 1640 medium (#11835030; Gibco) with 0.5% BSA) to 5 × 10^5^ cells per mL. These neutrophil samples were incubated with abN48-IgG1, abN48-2-IgG1, isotype antibody, CXCR1/2 inhibitor Navarixin (MK7123, #HY-10198, MedChemExpress) at different concentrations indicated in Fig. 4 in chemotaxis medium for 30 min at 37 °C. The chemoattractant, recombinant human IL-8 (#Z03262-25, GenScript) was contained in 600 μL chemotaxis medium in each lower chamber at concentration of 10 nM. A total of 250 μL of neutrophil suspension was loaded into each upper chamber and incubated for 60 min at 37 °C. After incubation, cells in the chemotaxis media in lower chambers were counted by hemocytometer (#717810, BRAND).

### Crystallization and data collection

pepN9-19 was synthesized by Sangon Biotech and its purity and identity validated by HPLC and mass spectrometry. The abN48-2 Fab was generated by papain (Sigma-Aldrich, at 1:50 w/w ratio) cleavage of abN48-2-IgG1 (lambda type) at 4 °C overnight in PBS buffer pH 7.4. Following cleavage, abN48-2 Fab was loaded onto a HiTrap lambda HP column (GE Healthcare) and eluted with 0.1 M sodium acetate at pH 3.0. The elution fractions were pooled and immediately applied to a Superdex200 increase 10/300GL gel filtration column (GE Healthcare) equilibrated with buffer containing 20 mM Tris pH 8.0 and 50 mM NaCl. Fractions containing purified Fab from the gel filtration column were then pooled and mixed with pepN9-19 at a molar ratio of 1:3. The mixture was incubated overnight at 4 °C before being further concentrated. The abN48 Fab and pepN9–19 complex was assembled following a similar protocol.

Crystals of abN48-2 with pepN9–19 were obtained at 18 °C using the hanging drop, vapor diffusion method. Diffraction quality crystals were grown on a siliconized cover slip by mixing 1 μL protein solution (15 mg mL^−1^) with 1 μL reservoir solution (0.1 M HEPES pH 7.5, 25% w/v polyethylene glycol 3350). Crystals of abN48 Fab with pepN9-19 were obtained in 0.1 M HEPES sodium pH 7.5, 2% v/v polyethylene glycol 400, 2.0 M ammonium sulfate. Crystals were flash-cooled in liquid nitrogen for data collection after adding 20% glycerol as cryo-protectant. Diffraction data were collected at beamline BL19U1 of the Shanghai Synchrotron Radiation Facility (SSRF) at a wavelength of 0.9789 Å and processed with the HKL3000 program^64^.

### Structure determination and refinement

The crystal structure of abN48-2 Fab in complex with pepN9-19 was solved by molecular replacement with the Phaser program in PHENIX^65^. The Fab search model was generated by combining a V_H_ domain built with SWISS-MODEL^66^ with a V_L_ domain from the 4E10 Fab structure (PDBID: 4XCN, chain L). The initial models were further improved by cycles of manual building and refinement using COOT^67^ and Refmac5 in CCP4i^68^. The quality of the final models was analyzed with MolProbity^69^. A summary of data collection and refinement statistics is outlined in Supplementary Table 2. Figures were prepared using program PyMol (The PyMOL Molecular Graphics System, Version 2.1 Schrödinger, LLC). Electrostatic calculations were performed with PDB2PQR^70^.

### Hydrogen–deuterium-exchange mass spectrometry

Amide hydrogen exchange of pepN48 alone was initiated by diluting 1 μL pepN48 at 50 μM into 19 μL D_2_O buffer (25 mM Tris, pD 8.0, 150 mM NaCl, 1 mM TCEP) at 10 °C. At different time points (0 s, 10 s, 30 s, 60 s, and 120 s), the labeling reaction was quenched by addition of chilled quench buffer (400 mM KH_2_PO_4_/K_2_PO_4_, pH 2.2, 50 mM TCEP) and immediately frozen in liquid nitrogen. For HDX-MS of pepN48 in the presence of abN48 or abN48-2, 1 μL pepN48 at 50 μM was first mixed with 1 μL abN48 or abN48-2 at 67 μM. The mixture was then labeled for 0, 10, 30, 60, or 120 s by adding 18 μL D_2_O buffer before being quenched and flash frozen. All frozen samples were stored at −80 °C until analysis.

The thawed samples were immediately injected into HPLC-MS (Agilent 1100) system equipped with in-line peptic digestion and desalting. The desalted digests were then separated with a Hypersil Gold^TM^ analytical column (Thermo) over an 18 min gradient and directly analyzed with an Orbitrap Fusion mass spectrometer (Thermo). The HPLC system was extensively cleaned with blank injections between samples to minimize any carryover. Peptide identification was performed by tandem MS/MS under orbi/orbi mode. All MS/MS spectra were analyzed using the MASCOT program, and final PSMs were filtered with a FDR of 1%. The initial analysis of the peptide centroids was carried out with HD-Examiner v2.4 (Sierra Analytics) and every peptide was then manually verified to check retention time, charge state, m/z range, and the presence of overlapping peptides. The peptide coverage of pepN48 was found to be 100% and the relative deuteration levels (%D) of each peptide was automatically calculated by HD-Examiner with the assumption that a fully deuterated sample retains 95% D (pepN48 alone) and 90% D (with abN48 or abN48-2) in current LC setting.

### *h*CXCR2 knock-in mice

A *h*CXCR2 transgenic mouse strain (C57BL/6) was constructed by replacement of the coding DNA sequence (CDS) of *m*CXCR2 in the genome with that of *h*CXCR2 via an embryonic stem (ES) cell gene targeting strategy as a previously described protocol^71^. Briefly, *h*CXCR2 CDS on a targeting vector was transduced into isolated mouse ES cells and replaced *m*CXCR2 CDS by recombination. Positive ES cells were selected against a neomycin marker and then injected into blastocysts, followed by transfer into a pseudopregnant foster mother. Chimeric offspring were selected for cross-breeding to obtain *h*CXCR2 homozygote mice. Homozygote mice were expanded via inbred breeding. Genotyping of the *h*CXCR2 mice was carried out via PCR with *h*CXCR2 or *m*CXCR2 specific primers followed by Sanger sequencing. Phenotyping of the *h*CXCR2 mice was carried out via staining of circulating cells with *h*CXCR2 or *m*CXCR2 specific antibodies. The mice were housed in a specific pathogen-free facility (5 mice per cage) with a light-dark cycle of (12–12) hours, temperature of (20–26) °C, and humidity of (40–70)%. The animal study was approved by the Institutional Animal Care and Use Committee of Shanghaitech University.

### Pharmacokinetic studies

A standard conversion curve of ELISA signal *vs*. serum concentration of abN48-2-IgG1 was first established by 1:2 serial dilution of a 1.25 μg mL^−1^ stock solution of abN48-2-IgG1 in *h*CXCR2 knock-in mouse serum. Sample mixtures were then 1:10 diluted into a PBS buffer supplemented with 0.5% BSA for ELISA assays. Concentrations of abN48-2-IgG1 in the serum samples and their corresponding ELISA readouts were plotted. The detection readout showed a linear correlation with the antibody concentration in the range of 0.6–78 ng mL^−1^ with a slope of 0.67.

For the PK study of abN48-2-IgG1, each of the *h*CXCR2 knock-in mice of 6–8 weeks old with body weight of about 20 g received a single i.v., s.c., or i.p. injection of 6.75 mg·kg^−1^ abN48-2-IgG1 antibody. Four mice were included in each injection group. Blood samples of 100 μL per mouse were collected from the periorbital sinus. Two of the 4 mice in each group were sampled at 15 min, 3 h, 24 h, and 72 h post-injection, while the remaining 2 mice in each group were sampled at 1 h, 12 h, and 48 h post-injection. Serum concentration of abN48-2-IgG1 in each sample was measured by ELISA and quantitated using the above standard curve. PK parameters were estimated by fitting the time courses into a single compartment model using WinNonlin. The parameters calculated included the area under the curve (AUC) of time-courses for serum concentrations from time 0 to the last measurable concentration (AUC_0-t_) and time 0 to infinity (AUC_0-inf_), clearance (CL), volume of distribution at steady-state (V_ss_), time to reach maximum concentration (T_max_), terminal half-life (t_1/2_), maximum plasma concentration (C_max_), etc.

### EAE model studies

The EAE model was induced in both wild type and *h*CXCR2 knock-in mice using synthetic myelin oligodendrocyte glycoprotein peptide (MOG_35-55_, MEVGWYRSPFS RVVHLYRNGK; GenScript). Briefly, mice were *s.c*. injected with an emulsion of 100 μg MOG_35-55_ peptide mixed with complete Freud’s adjuvant (IFA, #vac-ifa, InvivoGen) containing 400 μg Mycobacterium tuberculosis H37 Ra (#231141, Difco). A total of 400 ng pertussis toxin (#180, List Biological) were *i.p*. injected on the same day of MOG_35-55_ immunization and a second time at 48 h later.

To evaluate the efficacy of abN48-2-IgG1, 7.5 mg kg^−1^ or 0.075 mg kg^−1^ of abN48-2-IgG1 was *i.p*. injected *q.4d*. from the day of PTX injection for a total of 3 injections. Vehicle (PBS) and negative (Fc) controls were administrated according to the same dosing schedule as that for the antibody. Negative (Fc) controls were administrated with the dose of 7.5 mg kg^−1^. SB225002, a small molecule allosteric inhibitor of *h*CXCR2 was employed as a benchmark. SB225002 was shown as a CXCR2-selective inhibitor, whose binding affinity to CXCR1 was 150-folds lower than that to CXCR2. SB225002 is widely used in in vivo studies of CXCR2 related neutrophil trafficking responsive to inflammation, especially some studies in the EAE models^52,54,55^. In the current experiment, 0.5 mg kg^−1^ SB225002 was *i.p*. administrated once every day throughout the EAE experiment (from Day 1 to Day23). Each cohort group contained 4 mice. Clinical scores were recorded according to a 5-point scoring system (0, no clinical symptoms; 0.5, Tip of tail is limp; 1, Limp of the whole tail; 2, Limp tail and weakness of hind legs; 3, Limp tail and complete paralysis of hind legs; 4, Complete hind leg and partial front leg paralysis; 5, Dead. Mouse is euthanized due to severe paralysis.).

To analyze the effect of abN48-2-IgG1 on peripheral neutrophils, blood samples were collected 24 h after the first injection of the antibody and subjected to FACS analyses (see “Flow-cytometry” above). All animal use and experiments were approved by the Institutional Animal Care and Use Committee at Shanghaitech University.

### Statistical methods

All statistical tests were performed with Graphpad Prism 7 software. Values of measurements were expressed as mean with error bar of standard deviation (s.d.) in figures. Numbers to get the mean (*n* value) were chosen according to assay features and indicated in each figure legend. For statistical comparison, Dunnett’s multiple comparisons test of one-way ANOVA with Geisser-Greenhouse correction was employed. Consideration of significance by *p* values and exact *p* values were specified in legends.

### Reporting summary

Further information on research design is available in the Nature Research Reporting Summary linked to this article.

## Supplementary information

Supplementary Information

Reporting Summary

## Data Availability

Structural data have been deposited in the Protein Data Bank under accession codes 6KVA (abN48-2/pepN9-19) and 6KVF (abN48/pepN9-19). Source data are provided with this paper. All other data that support the findings of this manuscript are available from the corresponding authors upon reasonable request. Source data are provided with this paper.

## Source data

Source Data

## References

[1] Hauser AS, Attwood MM, Rask-Andersen M, Schioth HB, Gloriam DE (2017). Trends in GPCR drug discovery: new agents, targets and indications. Nat. Rev. Drug Disco..

[2] Fredriksson R, Lagerstrom MC, Lundin LG, Schioth HB (2003). The G-protein-coupled receptors in the human genome form five main families. Phylogenetic analysis, paralogon groups, and fingerprints. Mol. Pharmacol..

[3] Gilman AG (1987). G proteins: transducers of receptor-generated signals. Annu Rev. Biochem..

[4] Rajagopal S, Rajagopal K, Lefkowitz RJ (2010). Teaching old receptors new tricks: biasing seven-transmembrane receptors. Nat. Rev. Drug Disco..

[5] Markham A (2018). Erenumab: first global approval. Drugs.

[6] Kasamon YL (2019). FDA approval summary: Mogamulizumab-kpkc for mycosis fungoides and sezary syndrome. Clin. Cancer Res..

[7] Boshuizen RS (2014). A combination of in vitro techniques for efficient discovery of functional monoclonal antibodies against human CXC chemokine receptor-2 (CXCR2). MAbs.

[8] Bradley ME (2015). Potent and efficacious inhibition of CXCR2 signaling by biparatopic nanobodies combining two distinct modes of action. Mol. Pharmacol..

[9] Wu LJ (1996). Discrete steps in binding and signaling of interleukin-8 with its receptor. J. Biol. Chem..

[10] Rossant CJ (2014). Phage display and hybridoma generation of antibodies to human CXCR2 yields antibodies with distinct mechanisms and epitopes. MAbs.

[11] Huse WD (1989). Generation of a large combinatorial library of the immunoglobulin repertoire in phage lambda. Science.

[12] Barbas CF, Bain JD, Hoekstra DM, Lerner RA (1992). Semisynthetic combinatorial antibody libraries: a chemical solution to the diversity problem. Proc. Natl Acad. Sci. USA.

[13] Kaplon H, Reichert JM (2019). Antibodies to watch in 2019. MAbs.

[14] Lerner RA (2016). Combinatorial antibody libraries: new advances, new immunological insights. Nat. Rev. Immunol..

[15] Xie J, Zhang H, Yea K, Lerner RA (2013). Autocrine signaling based selection of combinatorial antibodies that transdifferentiate human stem cells. Proc. Natl Acad. Sci. USA.

[16] Qiang M (2018). Selection of an ASIC1a-blocking combinatorial antibody that protects cells from ischemic death. Proc. Natl Acad. Sci. USA.

[17] Blanchard JW (2017). Replacing reprogramming factors with antibodies selected from combinatorial antibody libraries. Nat. Biotechnol..

[18] Back M (2011). International Union of Basic and Clinical Pharmacology. LXXXIV: leukotriene receptor nomenclature, distribution, and pathophysiological functions. Pharm. Rev..

[19] Lane HC, Anand AR, Ganju RKCbl (2006). and Akt regulate CXCL8-induced and CXCR1- and CXCR2-mediated chemotaxis. Int Immunol..

[20] Sawant KV (2015). Chemokine CXCL1-mediated neutrophil trafficking in the lung: role of CXCR2 activation. J. Innate Immun..

[21] Borregaard N (2010). Neutrophils, from marrow to microbes. Immunity.

[22] Eash KJ, Greenbaum AM, Gopalan PK, Link DCCXCR2 (2010). and CXCR4 antagonistically regulate neutrophil trafficking from murine bone marrow. J. Clin. Investig..

[23] Metzemaekers M, Gouwy M, Proost P (2020). Neutrophil chemoattractant receptors in health and disease: double-edged swords. Cell Mol. Immunol..

[24] Kaur M, Singh D (2013). Neutrophil chemotaxis caused by chronic obstructive pulmonary disease alveolar macrophages: the role of CXCL8 and the receptors CXCR1/CXCR2. J. Pharm. Exp. Ther..

[25] Barnes PJ (2018). Targeting cytokines to treat asthma and chronic obstructive pulmonary disease. Nat. Rev. Immunol..

[26] Boppana NB (2014). Blockade of CXCR2 signalling: a potential therapeutic target for preventing neutrophil-mediated inflammatory diseases. Exp. Biol. Med (Maywood).

[27] Lerner CA, Lei W, Sundar IK, Rahman I (2016). Genetic ablation of CXCR2 protects against cigarette smoke-induced lung inflammation and injury. Front Pharm..

[28] Woodberry T, Bouffler SE, Wilson AS, Buckland RL, Brustle A (2018). The emerging role of neutrophil granulocytes in multiple sclerosis. J. Clin. Med..

[29] Stoolman JS, Duncker PC, Huber AK, Segal BM (2014). Site-specific chemokine expression regulates central nervous system inflammation and determines clinical phenotype in autoimmune encephalomyelitis. J. Immunol..

[30] Coffelt SB, Wellenstein MD, de Visser KE (2016). Neutrophils in cancer: neutral no more. Nat. Rev. Cancer.

[31] Lee YS (2012). Interleukin-8 and its receptor CXCR2 in the tumour microenvironment promote colon cancer growth, progression and metastasis. Br. J. Cancer.

[32] Dwyer MP, Yu YN (2014). CXCR2 receptor antagonists: a medicinal chemistry perspective. Curr. Top. Med. Chem..

[33] Dwyer MP, Yu Y (2014). CXCR2 modulators: a patent review (2009–2013). Expert Opin. Ther. Pat..

[34] Stadtmann A, Zarbock A (2012). CXCR2: From Bench to Bedside. Front Immunol..

[35] Hutchings CJ, Koglin M, Olson WC, Marshall FH (2017). Opportunities for therapeutic antibodies directed at G-protein-coupled receptors. Nat. Rev. Drug Discov..

[36] Larosa GJ (1992). Amino terminus of the interleukin-8 receptor is a major determinatnt of receptr subtype specificity. J. Biol. Chem..

[37] Gayle RB (1993). Importance of the amino terminus of the interleukin-8 receptor in ligand interactions. J. Biol. Chem..

[38] Katancik JA, Sharma A, de Nardin E (2000). Interleukin 8, neutrophil-activating peptide-2 and GRO-alpha bind to and elicit cell activation via specific and different amino acid residues of CXCR2. Cytokine.

[39] Katancik JA, Sharma A, Radel SJ, DeNardin E (1997). Mapping of the extracellular binding regions of the human interleukin-8 type B receptor. Biochem. Biophys. Res. Commun..

[40] Ahuja SK, Lee JC, Murphy PM (1996). CXC chemokines bind to unique sets of selectivity determinants that can function independently and are broadly distributed on multiple domains of human interleukin-8 receptor B. Determinants of high affinity binding and receptor activation are distinct. J. Biol. Chem..

[41] Luo ZW, Butcher DJ, Huang ZW (1997). Molecular modeling of interleukin-8 receptor beta and analysis of the receptor-ligand interaction. Protein Eng..

[42] Park SH, Casagrande F, Cho L, Albrecht L, Opella SJ (2011). Interactions of interleukin-8 with the human chemokine receptor CXCR1 in phospholipid bilayers by NMR spectroscopy. J. Mol. Biol..

[43] Park SH (2012). Structure of the chemokine receptor CXCR1 in phospholipid bilayers. Nature.

[44] Berkamp S, Park SH, De Angelis AA, Marassi FM, Opella SJ (2017). Structure of monomeric Interleukin-8 and its interactions with the N-terminal Binding Site-I of CXCR1 by solution NMR spectroscopy. J. Biomol. Nmr.

[45] Park SH, Berkamp S, Radoicic J, De Angelis AA, Opella SJ (2017). Interaction of monomeric interleukin-8 with CXCR1 mapped by proton-detected fast MAS solid-state NMR. Biophys. J..

[46] Sepuru KM, Nair V, Prakash P, Gorfe AA, Rajarathnam K (2020). Long-range coupled motions underlie ligand recognition by a chemokine. Receptor. iScience.

[47] Liu K (2020). Structural basis of CXC chemokine receptor 2 activation and signalling. Nature.

[48] Rose JJ, Foley JF, Murphy PM, Venkatesan S (2004). On the mechanism and significance of ligand-induced internalization of human neutrophil chemokine receptors CXCR1 and CXCR2. J. Biol. Chem..

[49] de Oliveira S, Rosowski EE, Huttenlocher A (2016). Neutrophil migration in infection and wound repair: going forward in reverse. Nat. Rev. Immunol..

[50] Kobayashi Y (2008). The role of chemokines in neutrophil biology. Front Biosci..

[51] Liu Y (2015). Preferential recruitment of neutrophils into the cerebellum and brainstem contributes to the atypical experimental autoimmune encephalomyelitis phenotype. J. Immunol..

[52] Inoue M (2016). An interferon-beta-resistant and NLRP3 inflammasome-independent subtype of EAE with neuronal damage. Nat. Neurosci..

[53] Liu L (2010). CXCR2-positive neutrophils are essential for cuprizone-induced demyelination: relevance to multiple sclerosis. Nat. Neurosci..

[54] Manjavachi MN (2010). The effects of the selective and non-peptide CXCR2 receptor antagonist SB225002 on acute and long-lasting models of nociception in mice. Eur. J. Pain..

[55] Kerstetter AE, Padovani-Claudio DA, Bai L, Miller RH (2009). Inhibition of CXCR2 signaling promotes recovery in models of multiple sclerosis. Exp. Neurol..

[56] Zweemer AJ, Toraskar J, Heitman LH, Ijzerman AP (2014). Bias in chemokine receptor signalling. Trends Immunol..

[57] Steen A, Larsen O, Thiele S, Rosenkilde MM (2014). Biased and g protein-independent signaling of chemokine receptors. Front Immunol..

[58] Handa S (1992). Concentrations of Interleukin-1b, Interleukin-6, Interleukin-8 and TNF-a; in Cerebrospinal Fluid from Children with Septic or Aseptic Meningitis. Kurum. Med. J..

[59] Yung MMH (2018). GRO-alpha and IL-8 enhance ovarian cancer metastatic potential via the CXCR2-mediated TAK1/NF kappa B signaling cascade. Theranostics.

[60] Holz O (2010). SCH527123, a novel CXCR2 antagonist, inhibits ozone-induced neutrophilia in healthy subjects. Eur. Respir. J..

[61] Xu L (2017). Design and characterization of a human monoclonal antibody that modulates mutant connexin 26 hemichannels implicated in deafness and skin disorders. Front Mol. Neurosci..

[62] Yang Z (2018). Affinity maturation of an TpoR targeting antibody in full-length IgG form for enhanced agonist activity. Protein Eng. Des. Sel..

[63] Hou S-C (2016). High throughput cytotoxicity screening of anti-HER2 immunotoxins conjugated with antibody fragments from phage-displayed synthetic antibody libraries. Sci. Rep..

[64] Minor W, Cymborowski M, Otwinowski Z, Chruszcz M (2006). HKL-3000: the integration of data reduction and structure solution–from diffraction images to an initial model in minutes. Acta Crystallogr D. Biol. Crystallogr..

[65] McCoy AJ (2007). Solving structures of protein complexes by molecular replacement with Phaser. Acta Crystallogr D. Biol. Crystallogr..

[66] Waterhouse A (2018). SWISS-MODEL: homology modelling of protein structures and complexes. Nucleic Acids Res..

[67] Emsley P, Cowtan K (2004). Coot: model-building tools for molecular graphics. Acta Crystallogr D. Biol. Crystallogr..

[68] Murshudov GN (2011). REFMAC5 for the refinement of macromolecular crystal structures. Acta Crystallogr D. Biol. Crystallogr..

[69] Chen VB (2010). MolProbity: all-atom structure validation for macromolecular crystallography. Acta Crystallogr D. Biol. Crystallogr..

[70] Dolinsky TJ, Nielsen JE, McCammon JA, Baker NA (2004). PDB2PQR: an automated pipeline for the setup of Poisson-Boltzmann electrostatics calculations. Nucleic Acids Res..

[71] Melton D. W. Gene-Targeting Strategies. In: *Transgenesis Techniques: Principles and Protocols* (ed Clarke A. R.). Springer New York (2002).

