# Supplementary material for: Selection of a picomolar antibody that targets CXCR2-mediated neutrophil activation and alleviates EAE symptoms
Source: Nat Commun. 2021 May 5;12:2547. doi: 10.1038/s41467-021-22810-z (PMC8100106; doi:10.1038/s41467-021-22810-z)
Supplement: Supplementary file 1 — Supplementary Information [file 41467_2021_22810_MOESM1_ESM.pdf]

Supplementary Information

Selection of a picomolar antibody that targets CXCR2-mediated neutrophil activation and alleviates EAE symptoms

Xiaojie Shi[1,†], Yue Wan[1,2,3,4,†], Nan Wang[1,2,3,4,†], Jiangchao Xiang[1,2,3,4,†], Tao Wang[1,2,3], Xiaofeng Yang[1], Ju Wang[1], Xuxue Dong[1,2,3], Liang Dong[1,2], Lei Yan[1], Yu Li[1,2,3], Lili Liu[1], Shinchen Hou[1], Zhenwei Zhong[1], Ian A. Wilson[6,7], Bei Yang[1], Guang Yang[1*], Richard A. Lerner[1,5*]

[1]Shanghai Institute for Advanced Immunochemical Studies, ShanghaiTech University, Shanghai 201210, China.

[2]School of Life Science and Technology, ShanghaiTech University, Shanghai 201210, China.

[3]University of Chinese Academy of Sciences, Beijing 100049, China.

[4]CAS Center for Excellence in Molecular Cell Science, Shanghai Institute of Biochemistry and Cell Biology, Chinese Academy of Sciences, Shanghai 200031, China.

[5]Department of Chemistry, The Scripps Research Institute, La Jolla, CA 92037, USA.

[6]Department of Integrative Structural and Computational Biology, The Scripps Research Institute, La Jolla, California 92037, USA.

[7]The Skaggs Institute for Chemical Biology, The Scripps Research Institute, La Jolla, California 92037, USA.

[†]These authors contributed equally: Xiaojie Shi, Yue Wan, Nan Wang, Jiangchao Xiang.

[*]e-mail: yangguang@shanghaitech.edu.cn (G.Y.); rlerner@scripps.edu (R.A.L.)

List of Extended data:

Supplementary Figures 1-11

Supplementary Tables 1-4

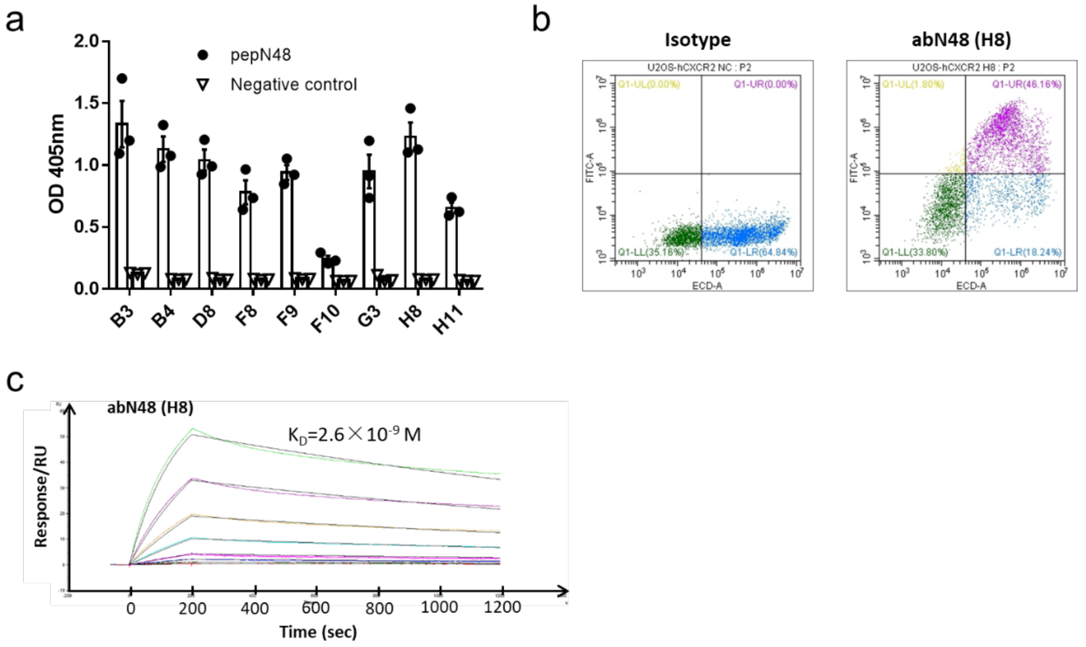

Supplementary Fig. 1 Selection of combinatorial antibody targeting *h*CXCR2. a, Nine positive clones from library panning were expressed as secreted combinatorial scFv antibodies, in which abN48 is represented here as H8. Binding of these clones with pepN48 was confirmed by ELISA. (n = 3 independent cell transfections which produce each clone of antibody, data presented as dots overlapped with mean (column) ± s.d. (error bars).) **b,** Binding of abN48 (H8) to *h*CXCR2 expressed on cell surface was measured by FACS. Isotype represents an irrelevant clone from another panning. **c,** Binding affinity of abN48 (H8) with pepN48 was determined by SPR assay on a Biacore T200. Curves in the sensorgram plot represent a range of concentrations of antibody: 0.1, 0.5, 1, 2, 5 and 10 nM.

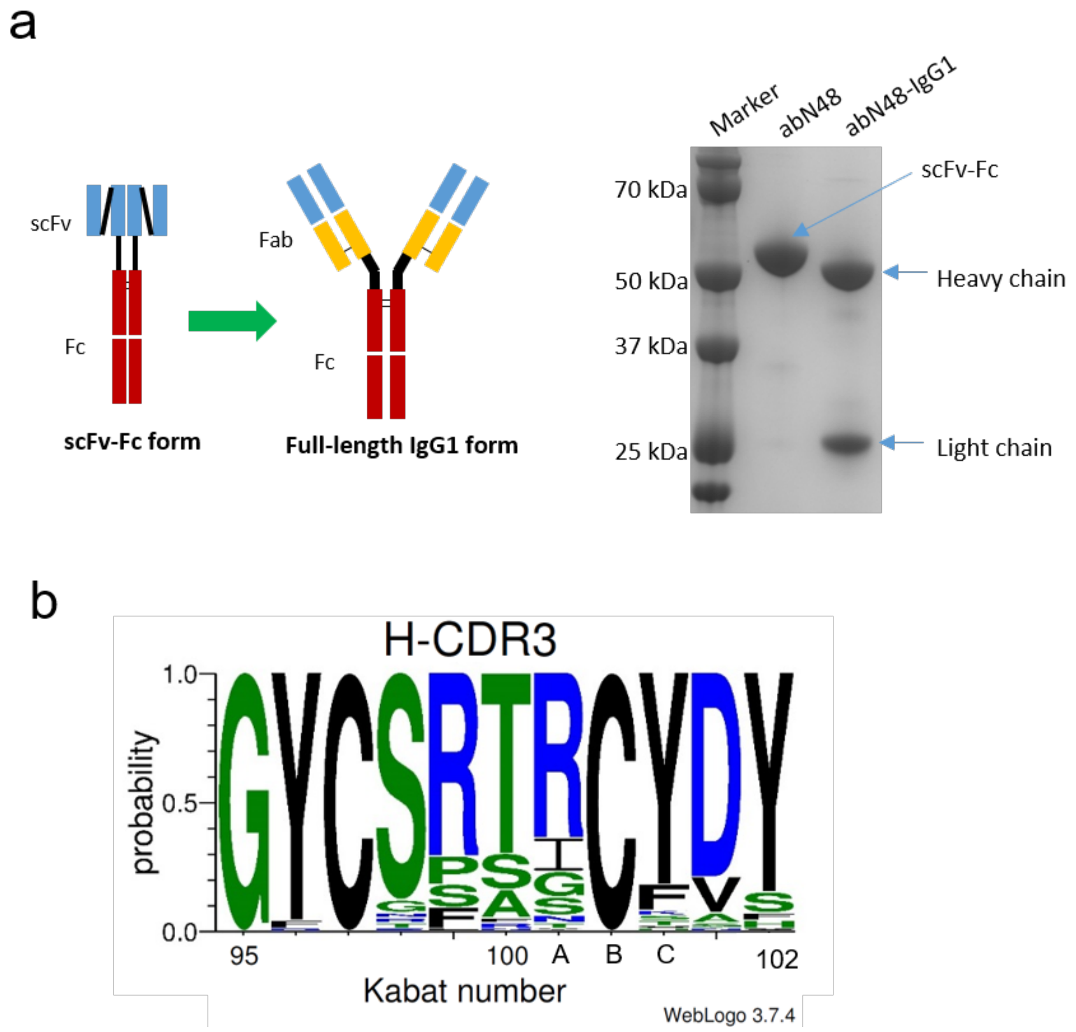

Supplementary Fig. 2 Full-length abN48-IgG1 purification and affinity-matured H-CDR3 sequence alignment. a, Illustration of full-length IgG1 format antibody construction from abN48 to abN48-IgG1, and SDS-PAGE of abN48 and abN48-IgG1. (SDS-PAGE is used for check of antibody expression and purification more than 3 independent batches, all showed consistent results) **b,** WebLogo analysis of different H-CDR3 sequences from affinity maturation. Amino acids are colored according to their chemical properties: polar-green, basic-blue, acidic-red and hydrophobic-black.

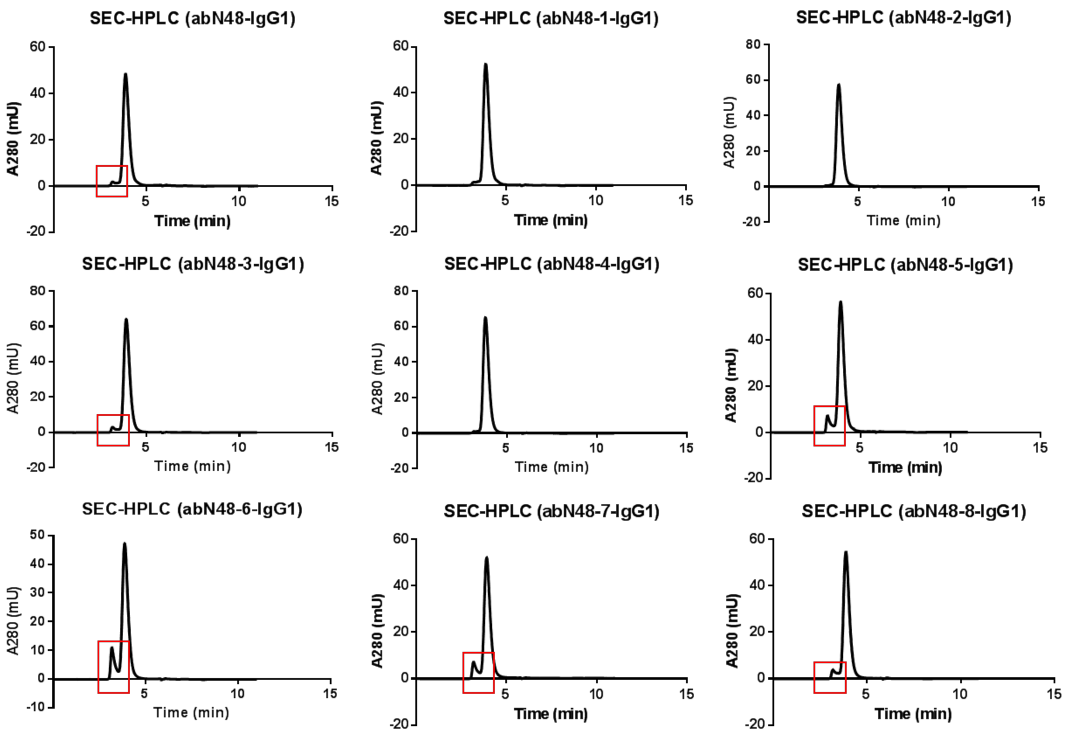

Supplementary Fig. 3 Homogeneity of recombinant antibodies by SEC-HPLC. SEC-HPLC chromatograms of abN48-IgG1 and its 8 analogues from affinity maturation: abN48-1-IgG1–abN48-8-IgG1. The shoulder peaks highlighted in red rectangular frames represent aggregates generated during incubation of the antibody at 42 ºC.

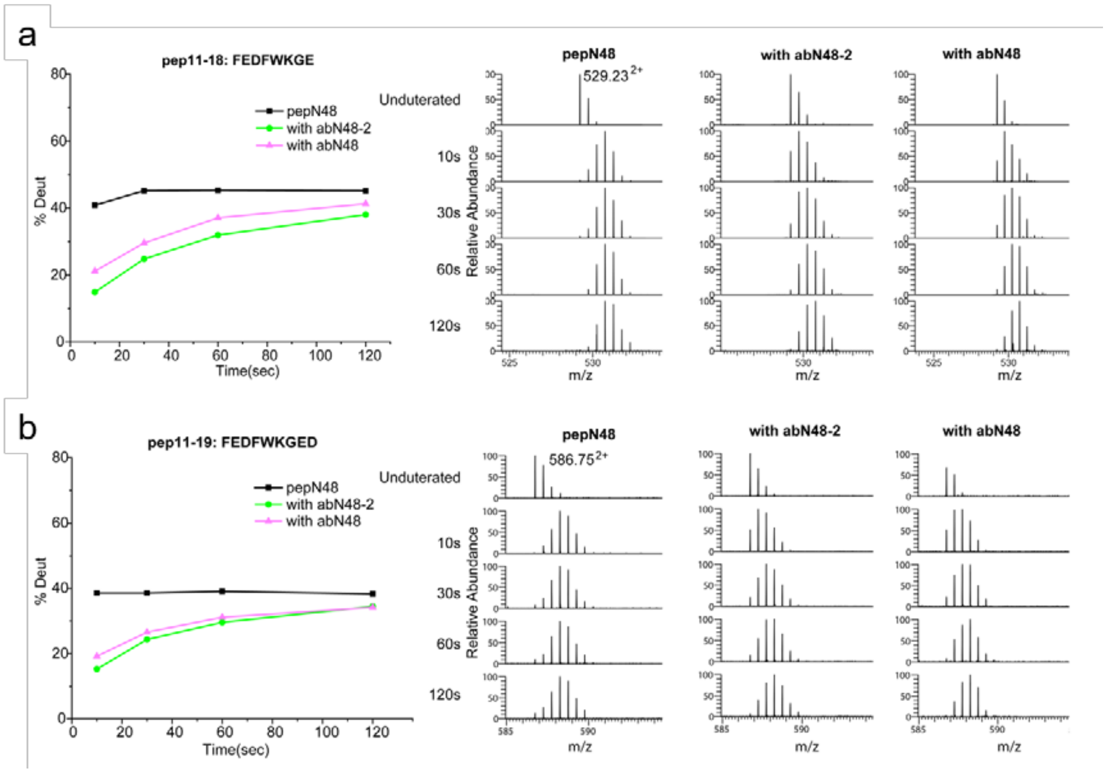

Supplementary Fig. 4 Deuterium uptake plots and mass spectra of indicated peptides from pepN48 in the absence and presence of abN48-IgG1 or abN48-2-IgG1. a, pep11-18 and **b**, pep11-19. Left: The deuterium uptake data are plotted as percent deuterium uptake versus time on a logarithmic scale. Right: Mass spectra of indicated peptides at different labeling time points, with the mass spectra of undeuterated samples shown as controls. The abN48 and abN48-2 in the graphs represent the corresponding IgG1 full-length antibodies.

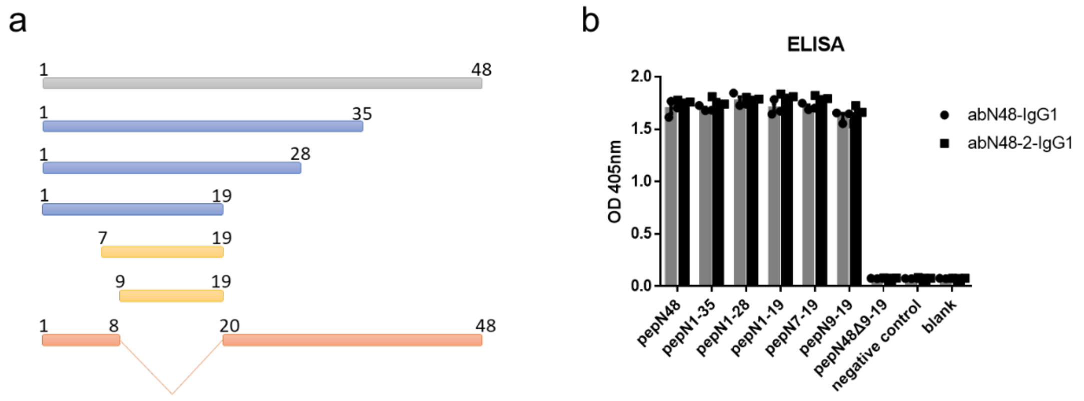

Supplementary Fig. 5 abN48 and abN48-2 epitopes on *h*CXCR2 as mapped by ELISA. a, Schematic illustration of designed N- or/and C-terminal truncated and 9-19 a.a. depleted synthetic peptides of *h*CXCR2 N-terminus. **b,** ELISA binding between abN48-IgG1 (grey) or abN48-2-IgG1 (black) and the peptides depicted in (**a**). (n = 6 ELISA wells coated with each corresponding antigen for each purified antibody, data presented as dots overlapped with mean (column) ± s.d. (error bars).)

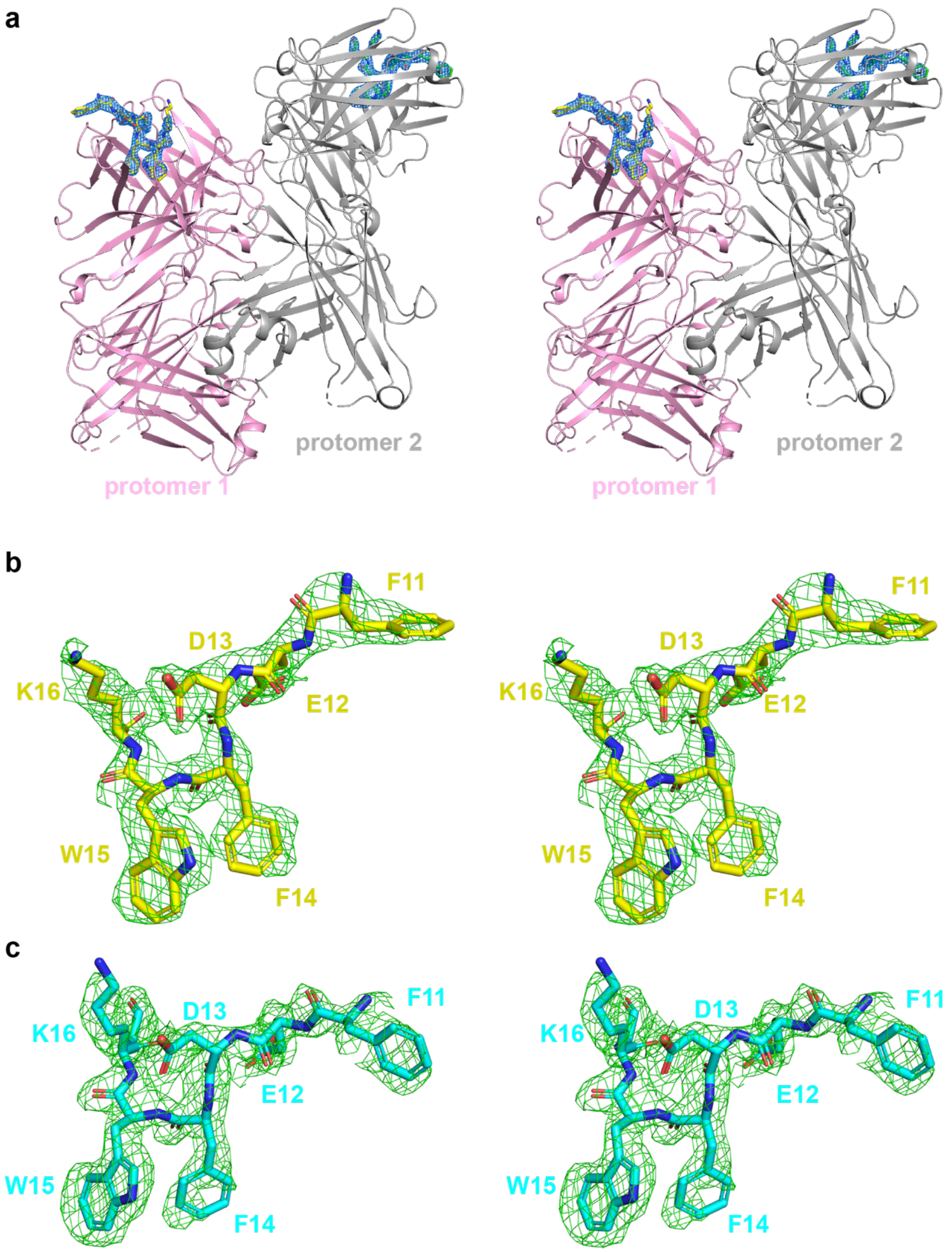

Supplementary Fig. 6 Overall structure of the abN48-2/pepN9-19 complex. a, The stereo view of abN48-2 /pepN9-19 complex. Two abN48-2 in one asymmetric unit of the crystal lattice are shown as cartoon models, and bound pepN9-19 peptides as stick models. One abN48-2 protomer is colored pink and the other colored grey.

b & **c,** In both protomers, residues 11-16 a.a. ('FEDFWK') of pepN9-19 could readily be traced in the Fo-Fc electron density omit map. pepN9-19 in protomer 1 is depicted as a yellow stick model and in protomer 2 as a cyan stick model. The peptide residues are labeled. The surrounding Fo-Fc omit map is colored green and contoured at 0.8 σ.

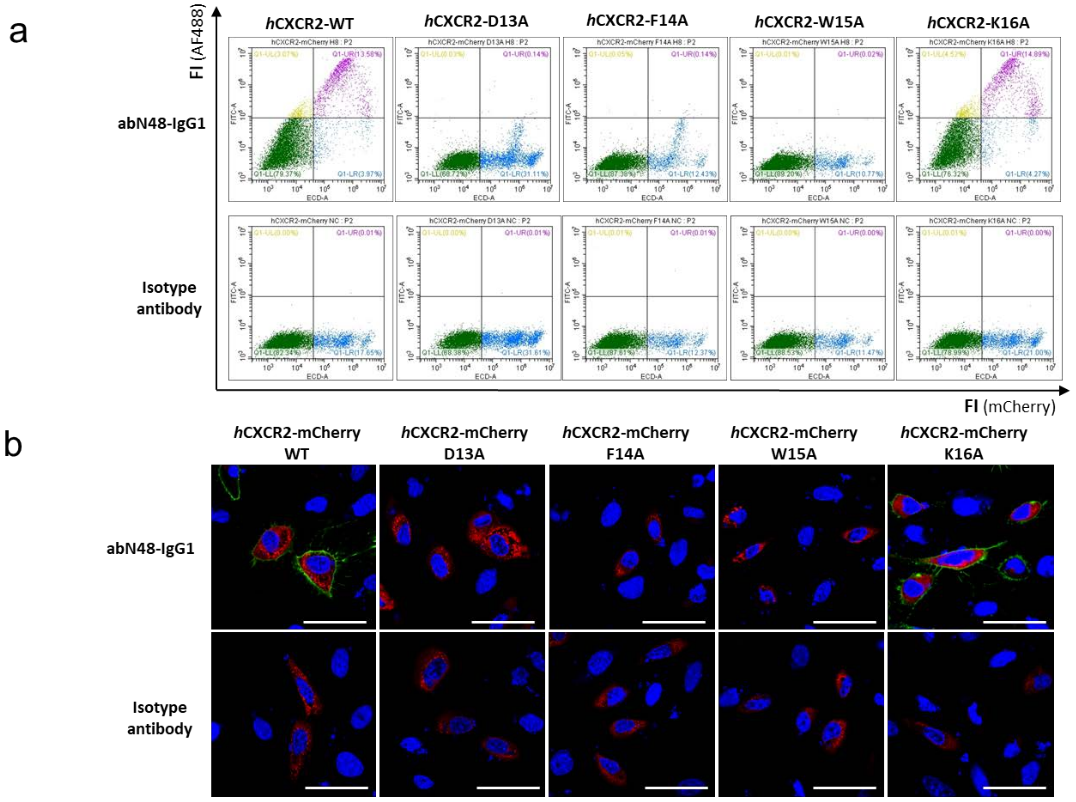

Supplementary Fig. 7 Site-directed mutagenesis for epitope mapping. The amino-acid residues in the IL8 binding region on *h*CXCR2 N-terminus (D13, F14, W15, K16) were subjected to alanine-scan, site-directed mutagenesis. The point mutants of *h*CXCR2 were ectopically expressed on U2OS cells as mCherry fusion proteins and stained with the abN48-IgG1 antibody. **a,** FACS and **b,** ICC representative results show that D13, F14 and W15 are the three key residues for antibody binding. An irrelevant antibody (isotype-matched antibody) is used as a control. White scale bars in panel b represent 50 µm. (3 independent experiments with several microscopic fields showed consistent results.)

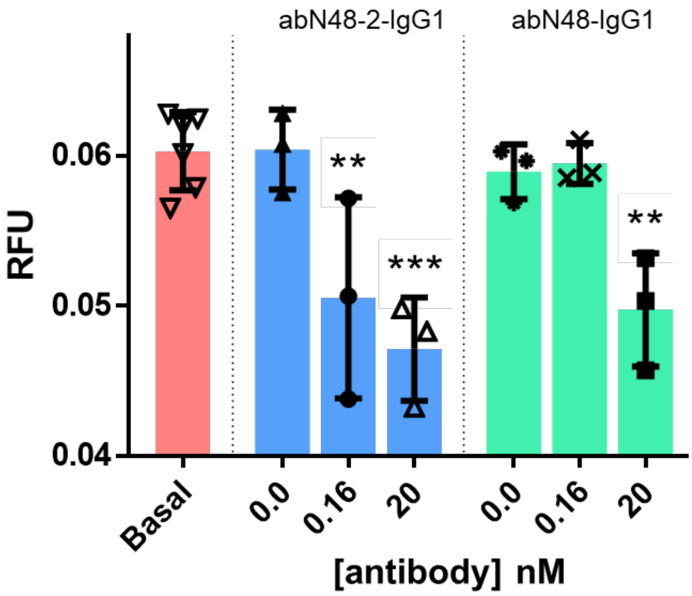

Supplementary Fig. 8 Inverse agonist effect of combinatorial antibody on CXCR2 mediated β-arrestin signaling. The intrinsic β-arrestin recruitment (represented as a red bar labeled as Basal) induced by the membrane expression of *h*CXCR2 was significantly reduced in the presence of different concentrations of abN48-IgG1 (green bars) and abN48-2-IgG1 (blue bars). (n = 3 biological samples in a single experiment, data presented as dots overlapped with mean (column) ± s.d. (error bars).) Each value of antibody (blue and green bars) was statistically compared with basal value (red bar) by one-way ANOVA Dunnett's multiple comparisons test. ** for $p < 0.01$ (adjusted $p = 0.0044$ and 0.0022 for 0.16 nM abN48-2-IgG1 and 20 nM abN48-IgG1 respectively), *** for $p < 0.001$ (adjusted $p = 0.0003$ for 20 nM abN48-2-IgG1).)

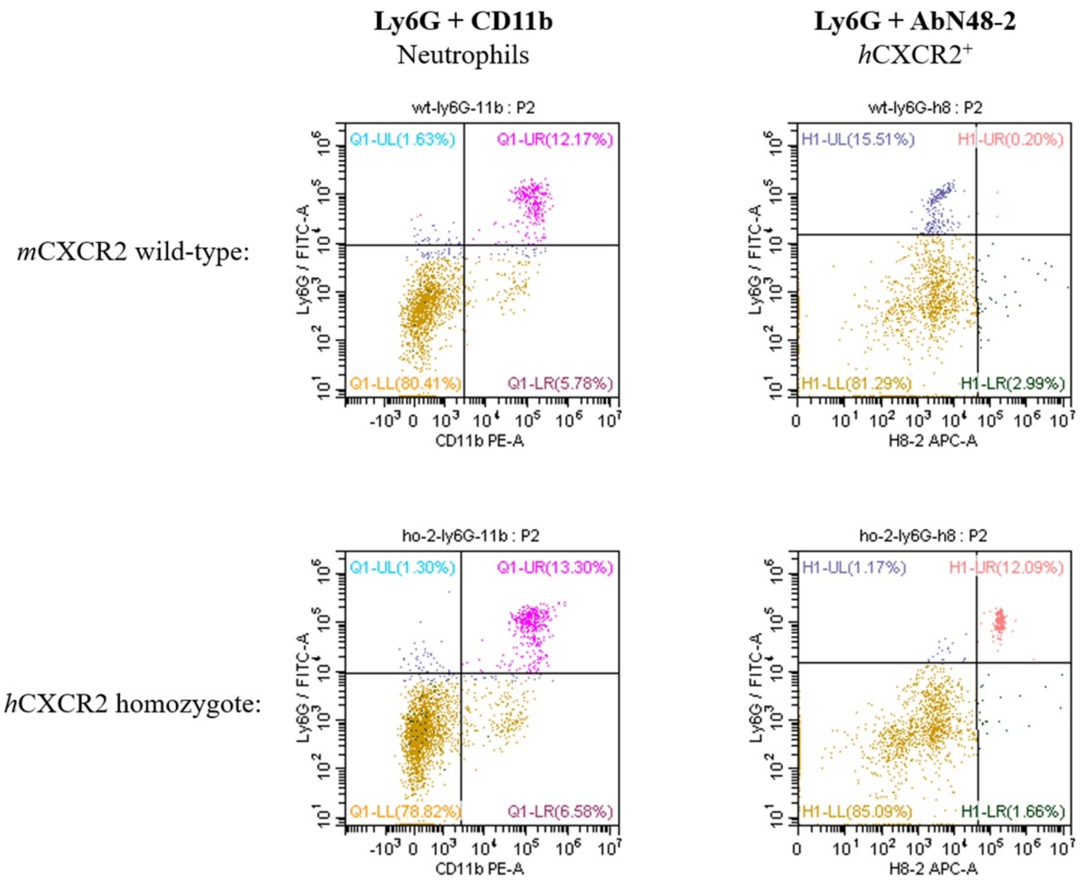

Supplementary Fig. 9 Phenotyping of circulating neutrophils in *h*CXCR2 mice. Circulating neutrophils in peripheral blood from *m*CXCR2 wild-type mice (up row) or *h*CXCR2 mice (bottom row) were determined by anti-Ly6G and anti-mCD11b antibodies (left column), while the *h*CXCR2$^+$ neutrophils were determined by anti-Ly6G and abN48-2-IgG1 (right column). (3 independent experiments and images showed consistent results.)

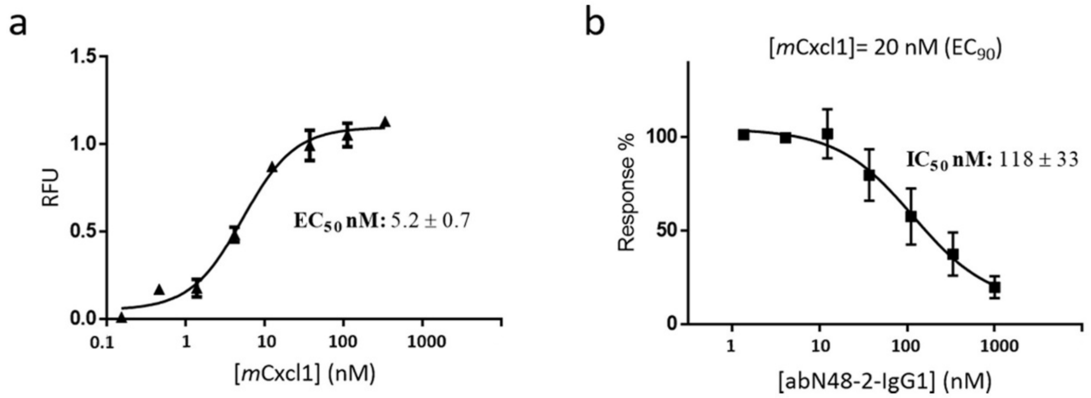

Supplementary Fig. 10 Inhibition of *m*CXCL1-*h*CXCR2 mediated Ca^{2+} signaling. **a,** *m*CXCL1 stimulation of *h*CXCR2-mediated Ca^{2+} flux. **b,** Inhibition of *m*CXCL1-*h*CXCR2 mediated Ca^{2+} influx (at EC_{90} concentration of mCxcl1) by abN48-2-IgG1. Data are represented as mean ± standard deviation. The calculated EC_{50} and IC_{50} values are listed next to the fitted curves. (n = 3 independent reporter cell samples measured in a single experiment. Each assay was repeated 3 times and all gave consistent results)

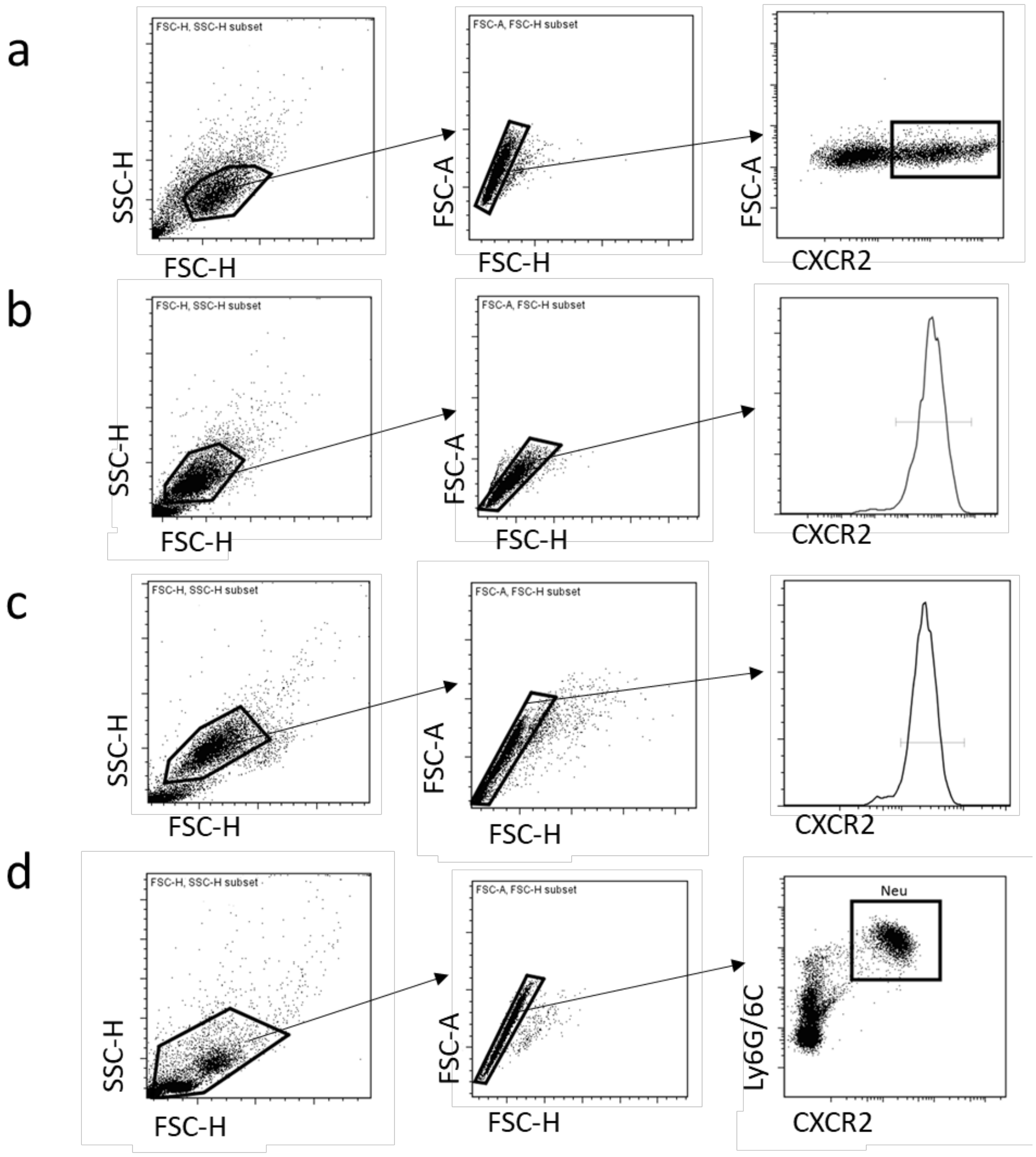

Supplementary Fig. 11 Gating strategies used for cell sorting. a, Gating strategy to sort CXCR2 positive cells from CXCR2-plasmids transfected cells. **b**, Gating strategy to sort CXCR2 positive cells from CXCR2-stable cell lines. **c**, Gating strategy to sort CXCR2+ neutrophils from purified neutrophil samples derived from peripheral bloods. **d**, Gating strategy to sort mouse neutrophils from mouse peripheral blood cells.

Supplementary Table 1. Kinetic parameters of abN48 and eight other new combinatorial antibodies selected by in vitro affinity maturation of H-CDR3. Data are displayed using exponential notation. Antibodies in the table represent the corresponding IgG1 full-length antibodies.

	k_{on} (1/Ms)	k_{off} (1/s)	K_D (M)
**abN48**	3.5E+5	3.5E-4	7.8E-10
**abN48-1**	2.5E+5	6.4E-5	2.6E-10
**abN48-2**	9.4E+5	1.7E-5	7.2E-12
**abN48-3**	3.6E+5	1.6E-4	6.3E-10
**abN48-4**	5.2E+5	1.6E-5	3.1E-11
**abN48-5**	8.1E+5	1.0E-6	1.2E-12
**abN48-6**	8.5E+4	1.8E-5	2.1E-10
**abN48-7**	1.3E+7	1.5E-4	1.2E-11
**abN48-8**	1.2E+6	2.0E-5	1.6E-11

Supplementary Table 2. Data collection and structure refinement statistics for antibody-peptide complexes

	abN48 Fab/pepN9-19	abN48-2 Fab/pepN9-19
**Data collection**		
Space group	*C* 1 2 1	*P* 1 2_1 1
Cell dimensions		
a, *b*, *c* (Å)	159.5, 76.1, 85.4	66.7, 96.3, 70.8
α, *β*, *γ* (°)	90, 108.5, 90	90, 109.9, 90
Resolution (Å)*	39.39-2.79 (2.89-2.79)	50.00-2.20 (2.28-2.20)
$R_{meas\ (\%)}$ *,a	11.3 (86.7)	10.6 (49.7)
$R_{pim\ (\%)}$ *,b	4.3 (33.4)	4.8 (24.0)
$CC_{1/2}$ *,c	1.0 (0.75)	1.0 (0.84)
$\langle I/\sigma(I)\rangle$ *	14.2(1.6)	12.9 (2.2)
Completeness (%)*	99.4 (98.3)	99.2 (94.2)
Redundancy*	6.7 (6.5)	4.7 (4.0)
**Refinement**		
Resolution (Å) *	39.39-2.79 (2.89-2.79)	48.51-2.20 (2.28-2.20)
No. Reflections*	24,073 (2,351)	42,397 (3,998)
R_{work} (%)*,d	21.0 (29.8)	18.8 (24.3)
R_{free} (%)*,e	26.8 (35.4)	23.1 (29.8)
No. atoms	6,478	7,022
Fab	6,378	6,458
pepN9-19	87	128
Ethanediol	/	8
Water	13	428
B-factors (Å^2)	61	34
Fab	61	33
pepN9-19	79	43
Ethanediol	/	38
Water	39	36
R.m.s. deviations		
Bond length (Å)	0.005	0.006
Bond angle (°)	1.08	1.09
Ramachandran plot (%)		
Favored	98.2	98.3
Allowed	1.8	1.7
Disallowed	0	0

*Statistics for the highest-resolution shell are shown in parentheses.

[a] $$R_{meas} = \frac{\sum_{hkl}[\frac{N(hkl)}{N(hkl)-1}]^{\frac{1}{2}} \times \sum_i |I_i(hkl) - \langle I(hkl)\rangle|}{\sum_{hkl}\sum_i I_i(hkl)} \quad (1)$$

[b] $$R_{pim} = \frac{\sum_{hkl}\left[\frac{1}{N(hkl)-1}\right]^{\frac{1}{2}} \times \sum_i |I_i(hkl) - \langle I(hkl)\rangle|}{\sum_{hkl}\sum_i I_i(hkl)} \quad (2)$$

[c] $$CC_{\frac{1}{2}} = \frac{\sum(x-\langle x\rangle)(y-\langle y\rangle)}{[\sum(x-\langle x\rangle)^2 \sum(y-\langle y\rangle)^2]^{\frac{1}{2}}} \quad (3)$$

[d] $$R_{work} = \frac{\sum\left||F_{obs}|-|F_{calc}|\right|}{\sum|F_{obs}|} \quad (4)$$

[e] R_{free} is defined as R_{work} calculated from 5% of the reflections that were excluded from refinement.

Supplementary Table 3. PK data of abN48-2-IgG1 in *h*CXCR2 transgenic mice.

Group	Mouse	T_{max} (h)	C_{max} (μg/mL)	AUC_{0-t} (μg·h/mL)	$AUC_{0-\infty}$ (μg·h/mL)	$t_{1/2}$ (h)	$MRT_{0-\infty}$ (h)	V_{ss} (L/kg)	CL (mL/min/kg)
ip	1	3.00	20.89	332.17	336.42	12.6	14.98		
	2	3.00	18.84	282.03	285.43	11.92	12.29		
iv	3	0.25	45.85	295.79	298.65	12.57	9.54	0.22	0.38
	4	0.25	34.27	312.79	315.17	9.51	9.53	0.20	0.36
sc	5	24.00	4.50	189.51					
	6	12.00	6.39	272.16					

Supplementary Table 4. List of primers used in this work.

Name	Sequence	Target	Orientation	Application
F1	GGTAAGACAAGAATCAGGGTGGCT	5'-UTR of mouse CXCR2	Forward	Genotyping
R1	TCAGCAGGAATACCAGGGCATAGAT	Human CXCR2 CDS	Reverse	Genotyping
F2	CATCAGGAGCAGGCTTAGAAGGCA	Before LoxP site	Forward	Genotyping
R2	CCTCCCTGCAAAACCAAGAATGACT	Before LoxP site	Reverse	Genotyping
VH-F1	ATTGCTTCAGTTTTAGCACTCGAG	Antibody heavy chain	Forward	Ab maturation
VH-R1	ACTCGCACAGTAATACACGGCC	Antibody heavy chain	Reverse	Ab maturation
VH-F2	GCCTACATGGAGCTGAGCAGGCTGA<br>GACCTGACGACACGGCCGTGTATTA<br>CTGTGCGAGTGGCTATTGTAGTAGTA<br>CCAGCTGCTATGACTACTGGGGCCA<br>GGGCACCCTGGTC *	H-CDR3	Forward	Ab maturation
VH-R2	ACCACCACTAGCCTGCAGACTAGT	Antibody heavy chain	Reverse	Ab maturation

* The sequence underlined is randomly mutated to generate degenerated primers, with 30% frequency randomly mutated to the other 3 bases.
