# Supplementary material for: Selection of a picomolar antibody that targets CXCR2-mediated neutrophil activation and alleviates EAE symptoms
Source: Nat Commun. 2021 May 5;12:2547. doi: 10.1038/s41467-021-22810-z (PMC8100106; doi:10.1038/s41467-021-22810-z)
Supplement: Supplementary file 2 — Reporting Summary [file 41467_2021_22810_MOESM2_ESM.pdf]

nature research

Corresponding author(s): Richard A. Lerner

Last updated by author(s): Mar 10, 2021

Reporting Summary

Nature Research wishes to improve the reproducibility of the work that we publish. This form provides structure for consistency and transparency in reporting. For further information on Nature Research policies, see our Editorial Policies and the Editorial Policy Checklist.

Statistics

For all statistical analyses, confirm that the following items are present in the figure legend, table legend, main text, or Methods section.

n/a	Confirmed	
☐	☒	The exact sample size (*n*) for each experimental group/condition, given as a discrete number and unit of measurement
☐	☒	A statement on whether measurements were taken from distinct samples or whether the same sample was measured repeatedly
☐	☒	The statistical test(s) used AND whether they are one- or two-sided<br>*Only common tests should be described solely by name; describe more complex techniques in the Methods section.*
☒	☐	A description of all covariates tested
☐	☒	A description of any assumptions or corrections, such as tests of normality and adjustment for multiple comparisons
☐	☒	A full description of the statistical parameters including central tendency (e.g. means) or other basic estimates (e.g. regression coefficient) AND variation (e.g. standard deviation) or associated estimates of uncertainty (e.g. confidence intervals)
☐	☒	For null hypothesis testing, the test statistic (e.g. *F*, *t*, *r*) with confidence intervals, effect sizes, degrees of freedom and *P* value noted<br>*Give P values as exact values whenever suitable.*
☒	☐	For Bayesian analysis, information on the choice of priors and Markov chain Monte Carlo settings
☐	☒	For hierarchical and complex designs, identification of the appropriate level for tests and full reporting of outcomes
☒	☐	Estimates of effect sizes (e.g. Cohen's *d*, Pearson's *r*), indicating how they were calculated

Our web collection on statistics for biologists contains articles on many of the points above.

Software and code

Policy information about availability of computer code

Data collection	Crystal diffraction data was collected and processed with the HKL3000 program.
Data analysis	The crystal structure was solved by molecular replacement with the Phaser program in PHENIX (v1.17.1). VH domain was built with SWISS-MODEL. The initial models were further improved using COOT (v0.9.1) and Refmac5 in CCP4i (v7.1). The quality of the final models was analyzed with MolProbity (v4.5.1). structure figures were prepared using program PyMol, Version 2.1. Electrostatic calculations were performed with PDB2PQR. MS/MS spectra were analyzed using the MASCOT program. The relative deuteration levels peptide in HDX was automatically calculated by HD-Examiner (v2.3). CytEpert (v2.3) was use for FACS analysis. PK parameters were estimated by curve fitting using WinNonlin (v6.0). Statistical tests were performed with Graphpad Prism (v7.00).

For manuscripts utilizing custom algorithms or software that are central to the research but not yet described in published literature, software must be made available to editors and reviewers. We strongly encourage code deposition in a community repository (e.g. GitHub). See the Nature Research guidelines for submitting code & software for further information.

Data

Policy information about availability of data

All manuscripts must include a data availability statement. This statement should provide the following information, where applicable:

- Accession codes, unique identifiers, or web links for publicly available datasets
- A list of figures that have associated raw data
- A description of any restrictions on data availability

Structure and data set in this work have been deposited in the Protein Data Bank with accession codes 6KVA and 6KVF. All other data that support the findings of this manuscript are available from the corresponding authors upon reasonable request.

Field-specific reporting

Please select the one below that is the best fit for your research. If you are not sure, read the appropriate sections before making your selection.

☒ Life sciences ☐ Behavioural & social sciences ☐ Ecological, evolutionary & environmental sciences

For a reference copy of the document with all sections, see nature.com/documents/nr-reporting-summary-flat.pdf

Life sciences study design

All studies must disclose on these points even when the disclosure is negative.

Sample size	According to the results of pre-experiments with 2-fold serial dilution of agonist stimulating signals in the signaling assays, the sample size of 3 is well to give significant differences (P<0.05) between groups. In the EAE experiment, sample size is chosen based on the experience of previously published work (such as Jones et al, 2018; Inoue et al, 2016; Liu et al, 2015; Eberle et al, 2015). No prior statistical sample size calculation is used.
Data exclusions	No data were excluded from the analyses.
Replication	For the representative results showed here including results in FACS, ICC, SPR, at least three repeats were carried out to ensure a consistence.
Randomization	Grouping of the mice in the EAE experiment is randomized. In the cell-based assays, cells were mixed and then randomly allocated to the experiment groups before various treatments including transfection.
Blinding	In the EAE experiment, the scoring persons were blind to the grouping information. Other data collections in this study are based on objective approaches, like reader machine.

Reporting for specific materials, systems and methods

We require information from authors about some types of materials, experimental systems and methods used in many studies. Here, indicate whether each material, system or method listed is relevant to your study. If you are not sure if a list item applies to your research, read the appropriate section before selecting a response.

Materials & experimental systems

n/a	Involved in the study	
☐	☒	Antibodies
☐	☒	Eukaryotic cell lines
☒	☐	Palaeontology and archaeology
☐	☒	Animals and other organisms
☒	☐	Human research participants
☒	☐	Clinical data
☒	☐	Dual use research of concern

Methods

n/a	Involved in the study	
☒	☐	ChIP-seq
☐	☒	Flow cytometry
☒	☐	MRI-based neuroimaging

Antibodies

Antibodies used	Anti-M13 HRP-conjugated secondary antibody (#27-9421-01, GE).<br>Anti-human Fc HRP-conjugated secondary antibody (#A0170, Sigma).<br>Anti-human IgG antibody (Promega, #W403B)<br>Alexa FluorTM 488 goat anti-human IgG (H+L) (#A11013, Invitrogen).<br>Alexa FluorTM 555 goat anti-human IgG (H+L) (#A21433, Invitrogen,).<br>CD11b Monoclonal Antibody (M1/70.15), PE (# RM2804, Invitrogen).<br>Ly-6G/Ly-6C Monoclonal Antibody (RB6-8C5), FITC (#11-5931-82, eBioscience).<br>Anti-mouse CD182 Antibody (REA942), APC (# 130-115-635, Miltenyi).<br>Anti-human CD182 antibody (REA208), FITC (#130-104-886, Miltenyi).<br>Alexa FluorTM 633 goat anti-human IgG (H+L) (#A-21091, Invitrogen).<br>Anti-chicken IgY, FITC (#A11039; Invitrogen).<br>Streptavidin Protein (# 21627, Invitrogen).<br>Anti-c-myc, chicken IgY fraction (# A21281, Life).<br>Dilution used for each antibody of each application is described in the methods section of the manuscript.
Validation	All antibodies were validated by the manufacturers or publications showed in the product sheets, briefly listed below:<br>Anti-M13 HRP-conjugated secondary antibody: Human,ELISA<br>Anti-human Fc HRP-conjugated secondary antibody: Human,WB/IHC/ELISA<br>Alexa FluorTM 488 goat anti-human IgG (H+L): Human, IHC/ICC/IF/FLOW<br>Alexa FluorTM 555 goat anti-human IgG (H+L): Human,IHC/ICC/IF

Anti-mouse CD182 Antibody-APC: Mouse, FLOW
Anti-human CD182-PE: Human, FLOW
Anti-mouse CD11B-PE (M1/70): Mouse, IHC/IF/FLOW
Anti-mouse Ly-6G/Ly-6C (Gr-1)-FITC (RB68C5): Mouse, IHC/ICC/IF/FLOW/IP/FN/ISH
Anti-chicken IgY-FITC, Invitrogen: Chicken,WB/IHC/ICC/IF/FLOW
Streptavidin Protein:Avidin, WB/IHC/ELISA
Anti-c-myc, chicken IgY fraction: Chicken, IHC/ICC/IF/FLOW

Eukaryotic cell lines

Policy information about cell lines

Cell line source(s)	HEK293T, cell bank of Chinese Academy of Science, Shanghai. HEK293F, Gibco. U2OS, cell bank of Chinese Academy of Science, Shanghai. Tango CXCR2-bla U2OS, Thermo. CHO-K1, Gibco.
Authentication	All cell lines were validated by STR check.
Mycoplasma contamination	All cell lines were tested negative for mycoplasma contamination
Commonly misidentified lines (See ICLAC register)	No commonly misidentified lines were used in this study.

Animals and other organisms

Policy information about studies involving animals; ARRIVE guidelines recommended for reporting animal research

Laboratory animals	Human CXCR2 transgenic C57BL/6 mice, female, 6~8 weeks. The condition that mice were maintained in is described in the manuscript.
Wild animals	No wild animal was used in this study.
Field-collected samples	No field-collected sample was used in this study.
Ethics oversight	All animal use and studies were approved by the Institutional Animal Care and Use Committee at Shanghaitech University.

Note that full information on the approval of the study protocol must also be provided in the manuscript.

Flow Cytometry

Plots

Confirm that:

☒ The axis labels state the marker and fluorochrome used (e.g. CD4-FITC).

☒ The axis scales are clearly visible. Include numbers along axes only for bottom left plot of group (a 'group' is an analysis of identical markers).

☒ All plots are contour plots with outliers or pseudocolor plots.

☒ A numerical value for number of cells or percentage (with statistics) is provided.

Methodology

Sample preparation	For CXCR2 binding assay, cell lines such as U2OS and 293T are transiently transfected with overexpresion plasmids and detected after 2days. For mouse peripheral lymphocytes analysis in EAE experiment, circulating blood was collected from orbital venous plexus, followed by red blood cell removal and wash with PBS twice.
Instrument	CytoFLEX, Beckman Coulter
Software	CytEpert (v2.3), Beckman Coulter
Cell population abundance	The transient transfection rate is above 40%. Whole lymphocytes excluding red blood cells were analyzed in the animal study.
Gating strategy	P1 and P2 were used to gate single live cells. Positive was gated based on the CXCR2-negative controls.

☒ Tick this box to confirm that a figure exemplifying the gating strategy is provided in the Supplementary Information.
